# Lansoprazole Upregulates Polyubiquitination of the TNF Receptor-Associated Factor 6 and Facilitates Runx2-mediated Osteoblastogenesis

**DOI:** 10.1016/j.ebiom.2015.11.024

**Published:** 2015-11-17

**Authors:** Kenichi Mishima, Hiroshi Kitoh, Bisei Ohkawara, Tatsuya Okuno, Mikako Ito, Akio Masuda, Naoki Ishiguro, Kinji Ohno

**Affiliations:** a Division of Neurogenetics, Center for Neurological Diseases and Cancer, Nagoya University Graduate School of Medicine, Nagoya, Aichi 466-8550, Japan; b Department of Orthopaedic Surgery, Nagoya University Graduate School of Medicine, Nagoya, Aichi 466-8550, Japan

**Keywords:** Runx2, Lansoprazole, Osteoblastogenesis, Drug repositioning, TRAF6, CYLD

## Abstract

The transcription factor, runt-related transcription factor 2 (Runx2), plays a pivotal role in the differentiation of the mesenchymal stem cells to the osteochondroblast lineages. We found by the drug repositioning strategy that a proton pump inhibitor, lansoprazole, enhances nuclear accumulation of Runx2 and induces osteoblastogenesis of human mesenchymal stromal cells. Systemic administration of lansoprazole to a rat femoral fracture model increased osteoblastogenesis. Dissection of signaling pathways revealed that lansoprazole activates a noncanonical bone morphogenic protein (BMP)-transforming growth factor-beta (TGF-β) activated kinase-1 (TAK1)–p38 mitogen-activated protein kinase (MAPK) pathway. We found by *in cellulo* ubiquitination studies that lansoprazole enhances polyubiquitination of the TNF receptor-associated factor 6 (TRAF6) and by *in vitro* ubiquitination studies that the enhanced polyubiquitination of TRAF6 is attributed to the blocking of a deubiquitination enzyme, cylindromatosis (CYLD). Structural modeling and site-directed mutagenesis of CYLD demonstrated that lansoprazole tightly fits in a pocket of CYLD where the C-terminal tail of ubiquitin lies. Lansoprazole is a potential therapeutic agent for enhancing osteoblastic differentiation.

## Highlights

• Lansoprazole facilitates osteoblastogenesis by upregulating Runx2, and accelerates fracture healing in rats.
• Lansoprazole activates the noncanonical BMP-TAK1-p38 MAPK pathway in mesenchymal stem cells.
• Lansoprazole enhances autopolyubiquitination of TRAF6 by tightly fitting in a pocket of a deubiquitination enzyme, CYLD.

Lansoprazole is a widely used proton pump inhibitor (PPI) for acid-related diseases such as gastroduodenal ulcers and reflux esophagitis. We found that lansoprazole facilitates differentiation and maturation of bone-forming cells, osteoblasts, and accelerates fracture healing in a rat model of femoral fracture. Lansoprazole fits in a pocket of an enzyme that breaks multi-ubiquitin chains, and inhibits its enzymatic activity. This inhibition activates a noncanonical bone morphogenetic protein (BMP)-mediated signaling and enhances osteoblastic differentiation in cultured cells and in a rat model of femoral facture. Lansoprazole is a potential therapeutic agent for fracture healing via upregulation of osteoblastogenesis.

## 1 Introduction

The major transcription factor runt-related transcription factor 2 (Runx2) plays a pivotal role in regulating chondroblast maturation and decision of the osteoblastic cell fate of the mesenchymal stem cells (MSCs) (Marie, 2008). The p38 mitogen-activated protein kinase (MAPK) and its upstream activator, transforming growth factor-beta (TGF-β) activated kinase-1 (TAK1), are crucial for the activation and, to a lesser extent, the expression of Runx2 in osteoblastogenesis (Greenblatt et al., 2010). In physiological cellular processes, TGF-β–bone morphogenetic protein (BMP) signaling transmits information through MAP kinase pathways by leveraging an adaptor molecule, TNF receptor-associated factor 6 (TRAF6), as well as canonical signaling molecules, Smads (Xie, 2013). TRAF6, which possesses the ubiquitin E3 ligase activity, is bound to TGF-β–BMP type I receptors and autopolyubiquitinated by itself. Unlike Lys48-linked polyubiquitination for proteasome-dependent protein degradation, TRAF6 catalyzes Lys63-linked polyubiquitination for intracellular signal transduction (Kulathu and Komander, 2012). The complex of TAK1–TAK1 binding protein (TAB) specifically recognizes TRAF6-conjugated Lys63-linked polyubiquitin chains and utilizes them as a scaffold for the assembly of TAK1 kinase complexes, which facilitates TAK1-mediated autophosphorylation of TAK1 itself and subsequent activation of downstream p38 MAPK (Sorrentino et al., 2008, Yamashita et al., 2008). Accordingly, Runx2 is upregulated through the TGF-β–BMP receptor (BMPR)–TRAF6–TAK1–p38 MAPK axis. Meanwhile, recent studies have witnessed emerging roles of the deubiquitination enzymes (DUBs) targeting to TGF-β–BMP type I receptors or some Smad molecules (Trompouki et al., 2003, Herhaus and Sapkota, 2014). One of the DUBs, cylindromatosis (CYLD), specifically catalyzes disassembly of Lys63-linked polyubiquitination and negatively regulates TGF-β–BMP and nuclear factor-kappa B (NF-κB) pathways.

The drug repositioning strategy, in which a drug currently used for a specific disease is applied to another disease, has gained increasing attention from both academia and industry in recent years (Abbott, 2002, Rothstein et al., 2005, Bian et al., 2009, Yamamoto et al., 2013, Matsushita et al., 2013, Takamatsu et al., 2014). The advantage of this strategy is that the identified drugs can be readily applied to clinical practice, because the optimal doses, adverse effects, and contraindications are already established. In this study, we identified by the drug repositioning strategy that lansoprazole, a proton pump inhibitor (PPI), upregulates Runx2 and induces osteoblastogenesis. We also proved the effect of lansoprazole in a rat model of femoral fracture in order to explore the possibilities of clinical application. We dissected the effect of lansoprazole and found that lansoprazole induces TRAF6 polyubiquitination, which then activates the noncanonical TAK1–p38 MAPK pathway and upregulates Runx2. Furthermore, *in silico* analysis predicted and site-directed mutagenesis revealed the binding of lansoprazole to a CYLD pocket and the inhibition of its deubiquitination activity by lansoprazole, which leads to enhanced polyubiquitination of TRAF6.

## 2 Materials and Methods

### 2.1 Cell Culture

We purchased mouse pluripotent mesenchymal C3H10T1/2 cells and human osteoblastic osteosarcoma (HOS) cells from Riken BioResource Center. We isolated primary bone marrow cells (BMCs) from 6-week-old male Sprague–Dawley (SD) rats as described previously (Takamine et al., 2002). We obtained human BMCs during surgery from 3 patients aged 9 years or younger with idiopathic acetabular dysplasia of the hip or osteonecrosis of the femoral head after appropriate written informed consent was given with prior approval by the ethical review committee of Nagoya University Graduate School of Medicine. We isolated monocyte-enriched fractions from the collected human BMCs by density gradient centrifugation with Ficoll-Paque (GE Healthcare) as described (Kitoh et al., 2004). Mesenchymal progenitors of rat and human were isolated by their binding ability to culture dishes. To seek for *ex vivo* applicability of lansoprazole in clinical settings, we also expanded human mesenchymal progenitors in StemPro (Gibco) medium. MSCs and mesenchymal progenitors were then subjected to differentiation in osteogenic medium containing 50 μg mL^− 1^ ascorbic acid, 10 mM β-glycerophosphate, and 10^− 7^ M dexamethasone. Additional information is provided in the Supplemental Materials and Methods.

### 2.2 Western Blot Analysis and Immunoprecipitation

Human MSCs were starved for 1 d in growth medium containing 1% fetal bovine serum (FBS) before addition of 20 μM lansoprazole. After 15 min of incubation, we added 100 ng mL^− 1^ recombinant BMP-2 for 0, 15, and 30 min. Cells were lysed in RIPA lysis buffer (Santa Cruz Biotechnology) supplemented with protease and phosphatase inhibitors. After centrifugation for 15 min at 18,000 ×* g*, supernatants were subjected to SDS-PAGE, followed by blotting onto PVDF membrane. We collected HOS cells after treatment with or without 20 μM lansoprazole for 2 d. We extracted nuclear and cytosolic fractions using the Nuclear Extraction kit (Cayman) according to the manufacturer's protocols. At the same time, we prepared a whole cell lysate by transferring a part of the cells to RIPA lysis buffer. For immunoprecipitation assay, collected HOS cells were lysed in passive lysis buffer containing 50 mM HEPES, 150 mM NaCl, 10% glycerol, 1% Triton X-100, 1.5 mM MgCl_2_, 1 mM EGTA, 100 mM NaF, and 10 mM sodium pyrophosphate supplemented with protease and phosphatase inhibitors, and incubated for 20 min, followed by centrifugation at 18,000 ×* g* for 15 min. After a two-fold dilution with the dilution buffer containing 50 mM HEPES, 150 mM NaCl, 0.1% Triton X-100, and 1 mM EDTA supplemented with protease and phosphatase inhibitors, immunoprecipitation was performed by incubation with 2 μg of antibody for overnight, followed by addition of Dynabeads Protein G to the diluted supernatant. The antibodies used are shown in the Supplemental Materials and Methods.

### 2.3 *In Cellulo* Protein Ubiquitination Assay Using Cultured Cells

HEK293 cells were transiently transfected with Flag-tagged human *TRAF6* cDNA in a CMV-based expression vector, which was a kind gift of Drs. Jun Ninomiya-Tsuji at North Carolina State University and Kunihiro Matsumoto at Nagoya University, using FuGENE 6 (Roche). After 1 d of culture, cells were subjected to complete serum starvation for 1 d, and then pretreated with or without lansoprazole for 30 min, followed by stimulation with or without 100 ng mL^− 1^ recombinant BMP-2 for the indicated time periods. For immunoprecipitation analysis, cells were washed twice, scraped in ice-cold PBS, and centrifuged at 18,000 ×* g* for 5 min. Non-covalent protein interactions were dissociated with 1% SDS and boiling for 5 min. Samples were diluted in PBS (1:10) containing 50 mM Tris–HCl, pH 7.5, 150 mM NaCl, 0.5% NP-40 supplemented with protease inhibitors, and centrifuged at 18,000 ×* g* for 15 min. Immunoprecipitation was performed by incubation with an anti-Flag M2 antibody (Sigma-Aldrich) overnight, followed by addition of Dynabeads Protein G (Invitrogen). The resultant immunoprecipitates were subjected to SDS-PAGE, followed by immunoblotting with antibodies against M2 Flag and ubiquitin to visualize TRAF6-associated polyubiquitin chains.

### 2.4 *In Vitro* Protein Ubiquitination Assay in a Test Tube

Human ubiquitin, ubiquitin-activating enzyme (E1), and UbcH5c (E2) were purchased from Abcam. Ubc13–Uev1a complex (E2) was purchased from Boston Biochem. Human wild-type *CYLD* cDNA (Kazusa DNA Research Institute) was cloned into pcDNA3.1(+) vector with a Flag tag at the C-terminal end. An R758A-single-mutant and an R758A and F766A-double-mutant Flag-tagged CYLD expression vectors were constructed using QuikChange site-directed mutagenesis kit (Stratagene). TRAF6 and CYLD were expressed in HEK293 cells and affinity-purified using anti-DYKDDDDK agarose beads (Wako Pure Chemical Ind.). Polyubiquitin chains were synthesized at 30 °C for 1 h in a reaction mixture containing 200 ng E1, 100 ng UbcH5c or Ubc13–Uev1a complex, 1–2 μg TRAF6, and 5 μg ubiquitin in buffer B (50 mM Tris–HCl pH 7.5, 10 mM MgCl_2_, 1.5 mM DTT, 5 mM ATP) with the indicated concentrations of lansoprazole. The reaction was terminated by addition of SDS sample buffer (Invitrogen), and subjected to immunoblotting analysis. For deubiquitination reaction, the polyubiquitination chains were synthesized as above but without lansoprazole and the reaction was terminated by adding 10 mM EDTA. The reaction mixture was subsequently pretreated with increasing amounts of lansoprazole for 15 min, followed by incubation with 100–200 nM CYLD in a reaction mixture containing 24 mM Tris–HCl pH 7.5 and 0.7 mM DTT at 30 °C for 1 h.

### 2.5 Animals

All animal care and handling conformed to the Regulations on Animal Experiments in Nagoya University and were approved by the Institutional Animal Care and Use Committee in Nagoya University. We purchased male Jcl:SD rats from CLEA Japan and made a rat model of left femoral fracture. Rats took 7.5 mg kg^− 1^ d^− 1^ of lansoprazole (Wako Pure Chemical Ind.) in drinking water, which was about 10 times more than the amount used for humans, and administration of the expected amount of lansoprazole was confirmed by monitoring water intake twice a week. For radiological analysis, 8-week-old rats were administered with lansoprazole or vehicle for 4 weeks (*n* = 6 per group). For histological analysis, ten-week-old rats were administered with lansoprazole or vehicle for 4 weeks (*n* = 8 per group). For analyzing an early phase of fracture repair, 8-week old rats were administered with lansoprazole or vehicle for 2 weeks (*n* = 4 per group). For analyzing a long-term effect of lansoprazole on intact metaphysis, unoperated rats were administered with lansoprazole or vehicle for twelve weeks (*n* = 4 per group). To label the mineralization front, we subcutaneously injected calcein (Dojindo, 8 mg per kg body weight) in 2% sodium hydrogen carbonate twice before being sacrificed.

### 2.6 Rat Model of Fracture Repair

All surgical procedures were conducted under sterile conditions. Rats were anesthetized by intraperitoneal injection of 30 mg kg^− 1^ sodium pentobarbital. A lateral incision on the left hindlimb was made to expose the shaft of femur. A 3-mm bone defect was created at the mid-shaft using a thin oscillating saw (NSK Dental LLC), and then periosteum was stripped circumferentially on both sides of osteotomy to prevent early bone union. Once a 1.2-mm diameter wire (Mizuho Corp.) was inserted from the cross-section into the proximal femoral canal in a retrograde manner, the both ends of osteotomy site were placed in contact with each other, and then the wire was reinserted across the osteotomy site into the distal femoral canal to apply stability. All rats were given prophylactic administration of sulfamethoxazole (50 mg kg^− 1^ d^− 1^, Sigma-Aldrich) and trimethoprim (10 mg kg^− 1^ d^− 1^, Sigma-Aldrich), and randomly assigned to receive either lansoprazole or vehicle. At 2 or 4 weeks after fracture, fractured femurs were extracted.

### 2.7 Radiological Assessment of Fracture Repair

At 1, 2, 3, and 4 weeks after fracture, lateral radiographs of the fractured hindlimb were made using a Multi soft X-ray film shooting apparatus (SOFTEX). Fracture union was defined by the formation of seamless bridging callus on at least one cortex on the lateral radiograph. After extracting femurs, bridging callus formation on 4 cortices was evaluated on the anteroposterior and lateral radiographs, and the number of seamless bridging calluses in the 4 cortices was counted (0 to 4). In addition, bridging callus area was measured on ImageJ software. Uniting (intramedullary) callus formation was determined by the disappearance of fracture line on the anteroposterior and lateral radiographs and its number was counted (0 to 2).

### 2.8 Bone Histological and Morphometrical Analyses

Bone specimens were sequentially dehydrated with 70% ethanol, 90% ethanol, and acetone. The specimens were then embedded without decalcification in methyl methacrylate. We prepared 6-μm sagittal sections of the whole femur and 30-μm transverse sections at the osteotomy sites with a microtome, and stained them with Villanueva Goldner stain. Bone parameters of the fracture sites were blindly evaluated by computer-assisted histomorphometry using Osteomeasure software (OsteoMetrics). For analysis of bone regeneration in the fracture sites, we compared the area of newly formed bone with that of granulation tissue, and calculated bone volume per granulation tissue volume (BV/TV) without setting definite region of interest (ROI) in the fracture sites. We summed bone histomorphometric values of sagittal and transverse sections to assess osteoblastogenesis in the fracture sites. In the fractured femurs, after excluding pin track areas in the distal femur, we defined the ROI in the metaphysis as the areas between 0.7 mm and 3.0 mm proximal to the growth plate in order to exclude the primary trabecular spongiosa. Cartilaginous callus area was measured using histological sections stained with alcian blue. Total callus area was defined as the sum of bridging callus area and anchoring callus area, and was measured by ImageJ software. Histological images were obtained using a BX51 (Olympus) with an UPlanFL N 20 × and 0.50 objective lens (Olympus) fitted with a DXC-390P camera (Sony) and a microscope M60 (Leica) with a 1.0 × objective lens fitted on a camera IC80 (Leica).

### 2.9 *In Cellulo* Osteoclast Formation

RAW 264.7 cells (Riken BioResource Center), a mouse macrophage-like cell line, were seeded onto a 96-well plate at a cell density of 2 × 10^5^ cells per well and cultured for 4 d with or without lansoprazole in the presence of receptor activator of NF-κB ligand (RANKL) (100 ng mL^− 1^; Peprotech). Tartrate-resistant acid phosphatase (TRAP)-positive cells with more than 3 nuclei were counted as osteoclasts.

### 2.10 *In Cellulo* Bone Resorption Assay

The pit formation assay was performed using a calcium phosphate-coated plate obtained from PG research (Kodaira, Japan). RAW 264.7 cells grown in α-MEM medium were cultured in a 48-well calcium phosphate-coated plate (5 × 10^3^ cells per well) with or without lansoprazole in the presence of RANKL (100 ng mL^− 1^) for 7 d. After removing the cells by washing the plate with 5% sodium hydrochloride solution, digital images of the resorption pits were captured. The pit area was measured following conversion of the original images to binary images with NIH ImageJ software.

### 2.11 Statistical Analysis

All data are presented as the mean and SD. We evaluated dose–responses using the Jonckheere–Terpstra trend test for the whole range of drug concentrations. Statistical difference between 2 groups was determined by the unpaired *t*-test. We considered a *p* value < 0.05 as statistically significant.

## 3 Results

### 3.1 Lansoprazole Upregulates Runx2

**Supplemental Fig. 1:**
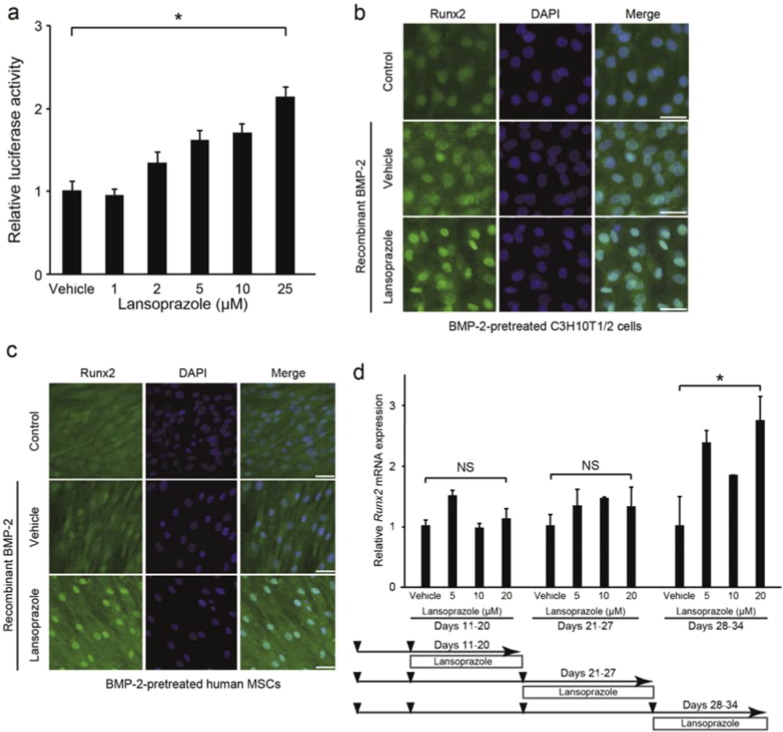
Effects of lansoprazole on the activity of human *RUNX2* P1 promoter, nuclear accumulation of Runx2 in mouse and human mesenchymal stromal cells, and expressions of *Runx2* gene in rat primary bone marrow cells. (a) Lansoprazole activates human *RUNX2* P1 promoter in a dose-dependent manner. Firefly luciferase activities of BMP-2-stimulated C3H10T1/2 cells that stably express pGL4-hRunx2P1-luc2 (C3H10T1/2-hRunx2P1 cells), in which the human *RUNX2* P1 promoter is fused to firefly luciferase cDNA. Luciferase activities are normalized to the mean of vehicle. Cells were treated with the indicated concentrations of lansoprazole for 1 d. *p < 0.001 by the Jonckheere–Terpstra trend test over the indicated concentration range of lansoprazole. (b) Runx2-specific immunofluorescence staining of C3H10T1/2 cells treated with lansoprazole for 3 d. Cells were pretreated with or without 100 ng ml^− 1^ of recombinant BMP-2 for 6 d. Scale bar, 50 μm. (c) Runx2-specific immunofluorescence staining of human MSCs treated with lansoprazole for 3 d. Cells were pretreated with or without 100 ng ml^− 1^ of recombinant BMP-2 for 3 d. Scale bar, 50 μm. (d) Lansoprazole upregulates the expression of Runx2 in rat bone marrow cells. Expression levels of endogenous *Runx2* mRNA by real-time RT-PCR in rat bone marrow cells. Cells were treated with lansoprazole for 2 d. The relative expression levels are normalized to the mean of vehicle. A sequential protocol for culturing cells is shown at the bottom. Lower arrowheads indicate cell splitting. *p = 0.012 (Days 28–34) by the Jonckheere–Terpstra trend test above the evaluated concentration ranges of lansoprazole. NS, not significant.

**Fig. 1:**
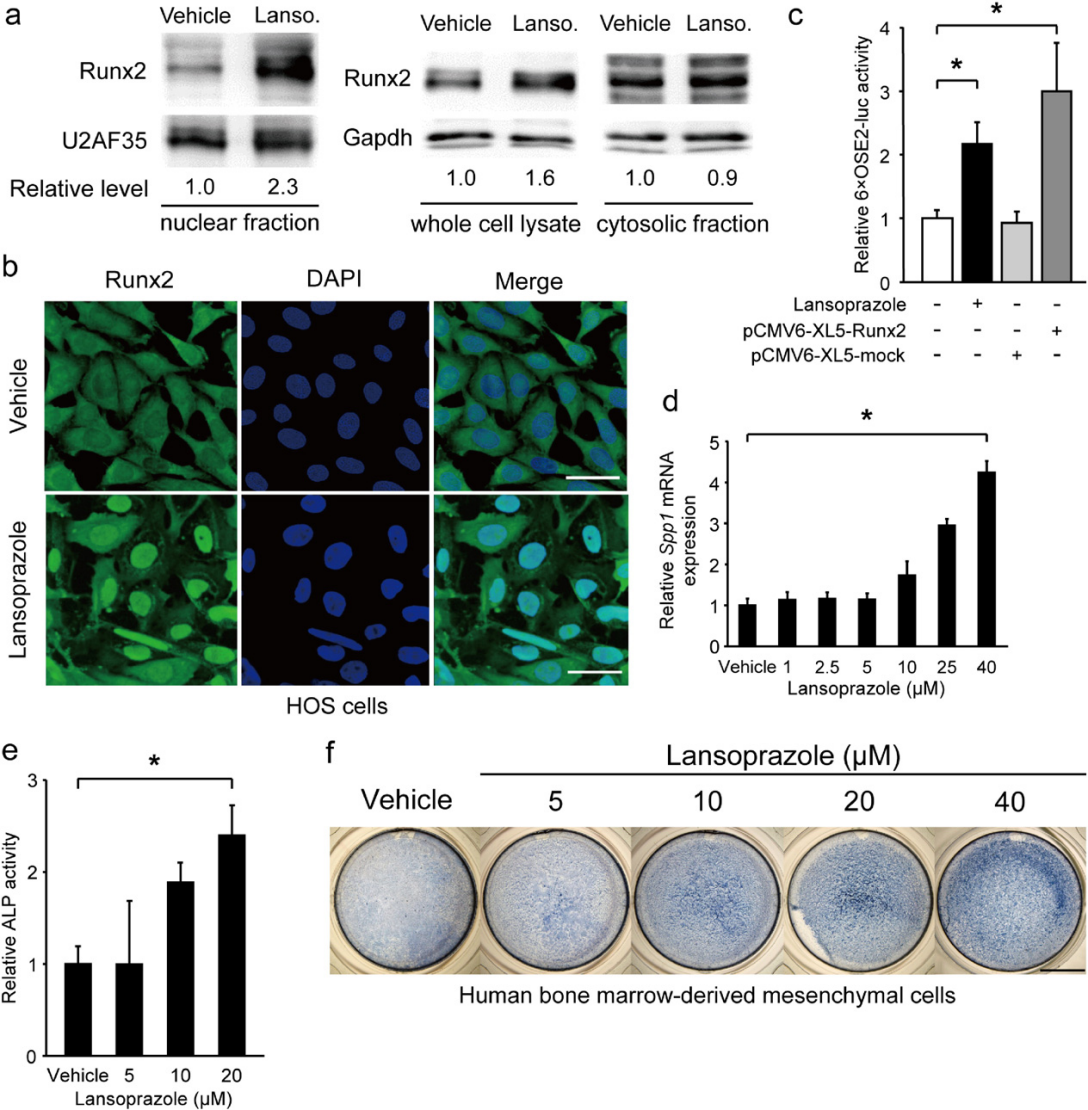
Lansoprazole induces nuclear accumulation of Runx2 and activates its transcriptional activity. (a) Immunoblotting of Runx2 in the nuclear and cytosolic fractions as well as in the whole cell lysate of HOS cells after adding 20 μM lansoprazole for 2 d. The relative expression levels were densitometrically estimated using an essential splicing factor U2AF35 for the nuclear fraction and Gapdh for the cytosolic fraction and the whole cell lysate as a control. Lanso., lansoprazole. (b) Runx2-specific immunofluorescence staining of HOS cells treated with lansoprazole for 3 d. Scale bar, 50 μm. (c) Activity of Runx2-responsive luciferase reporter vector (6xOSE2-luc) in C3H10T1/2 cells. Cells were exposed to 20 μM of lansoprazole for 1 d without adding BMP-2. pCMV6-XL5-Runx2, human *RUNX2* cDNA clone. (d) Expression levels of endogenous *Spp1* mRNA by real-time RT-PCR in C3H10T1/2 cells. Cells were pretreated with 100 ng mL^− 1^ of recombinant BMP-2 for 3 d, followed by incubation with the indicated concentrations of lansoprazole for 14 d. The relative expression levels were normalized to the mean of vehicle. (e) ALP activity of cell lysates of human bone marrow-derived mesenchymal cells. Cells were cultured in osteogenic medium for 19 d and lansoprazole was added from days 15 to 19. The relative activity was normalized to the mean of vehicle. (f) ALP staining of human bone marrow-derived mesenchymal cells, which were treated with the indicated concentrations of lansoprazole. Cells were cultured in osteogenic medium for 24 d and lansoprazole was added from days 18 to 24. Scale bar, 5 mm. In (c–e), the mean ± SD (*n* = 6) are indicated. *p < 0.0001 by unpaired *t*-test (c) and by the Jonckheere–Terpstra trend test over the indicated concentration range of lansoprazole (d and e). (d, e, and f) In the course of our analysis, 5–20 μM lansoprazole exhibits consistent dose–response effects, but data at concentrations less than 5 μM and more than 20 μM have not been omitted under the expectation that these concentrations will be of some help for future references.

**Fig. 2:**
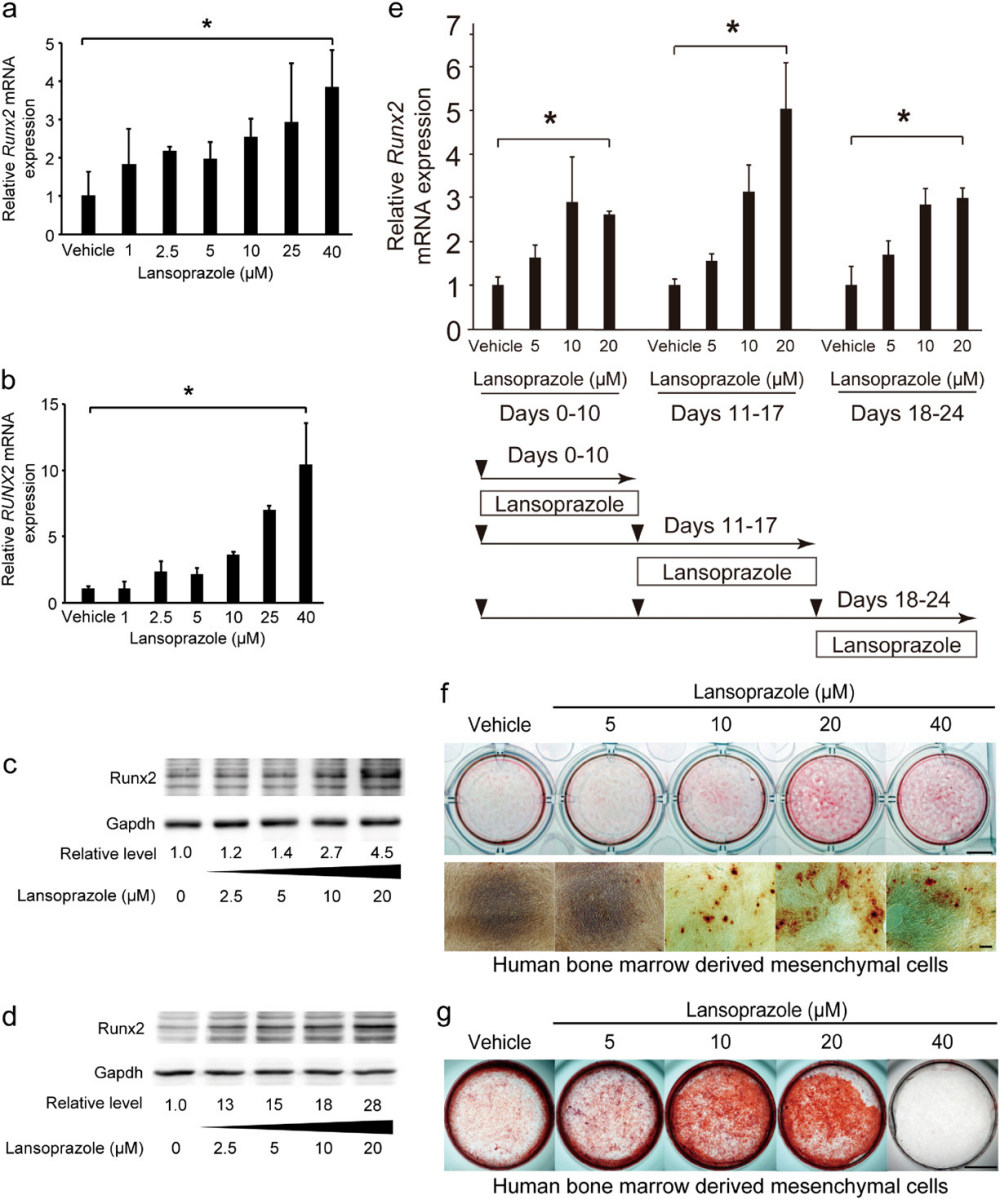
Lansoprazole upregulates the expression of Runx2 and matrix calcium deposition. (a) Expression levels of endogenous *Runx2* mRNA by real-time RT-PCR in BMP-2-pretreated C3H10T1/2 cells. Cells were treated with lansoprazole for 2 d. The relative expression levels were normalized to the mean of vehicle. (b) Expression levels of endogenous *RUNX2* mRNA by real-time RT-PCR in HOS cells. Cells were treated with lansoprazole for 2 d. The relative expression levels were normalized to the mean of vehicle. (c) Expression levels of endogenous Runx2 protein in BMP-2-pretreated C3H10T1/2 cells. Cells were treated with lansoprazole for 3 d. Densitometric values were normalized to that of Gapdh and also to vehicle. (d) Expression levels of endogenous Runx2 protein in HOS cells. Cells were treated with lansoprazole for 3 d. Densitometric values were normalized to that of Gapdh and also to vehicle. (e) Expression levels of endogenous *RUNX2* mRNA by real-time RT-PCR in human MSCs. The relative expression levels were normalized to the mean of vehicle. Sequential protocol of lansoprazole administration is indicated at the bottom. Lower arrowheads indicate the time points when we passaged cells. (f) Matrix calcium deposition by Alizarin red staining in human bone marrow-derived mesenchymal cells that were induced to differentiate immediately after isolation. Cells were cultured in osteogenic medium for 21 d and lansoprazole was added from days 15 to 21. Scale bars, 5 mm for dishes and 200 μm for magnified images. Note that the temporal protocol of human bone marrow-derived mesenchymal cells in (f) is different from that of human MSCs in (e), because the time courses of differentiation of these cells are different. (g) Matrix calcium deposition by Alizarin red staining in human bone marrow-derived mesenchymal cells that were induced to differentiate after proliferation for 23 d. Cells were proliferated in non-osteogenic medium for 23 d followed by additional culture in osteogenic medium up to day 44, and lansoprazole was added from days 24 to 44. In (f), cells were differentiated immediately after isolation, whereas in (g), cells were first proliferated for 23 d to increase the number of cells. Scale bars, 5 mm. In (a, b, and e), the relative expression levels were normalized to the mean of vehicle, and the mean ± SD (*n* = 6) are indicated. *p < 0.01 (a), *p < 0.0001 (b), *p < 0.01 (c, days 0–10), *p < 0.001 (c, days 11–17), and *p < 0.002 (c, days 18–24) by the Jonckheere–Terpstra trend test over the indicated concentration range of lansoprazole. In (c and d), the densitometric values were normalized to that of Gapdh and also to vehicle.

Runx2 is translocated into the nucleus where it binds to the cis-acting osteoblast-specific element 2 (OSE2) on promoters of the osteogenic genes including *Alpl* encoding alkaline phosphatase (ALP) and *Spp1* encoding osteopontin (Otto et al., 2003). We screened 1186 FDA-approved compounds for activating the human *RUNX2* P1 promoter in mouse pluripotent mesenchymal C3H10T1/2 cells and found that lansoprazole increased the *RUNX2* P1 promoter activity in a dose-dependent manner (Fig. S1a). We found that lansoprazole enhanced intranuclear accumulation of Runx2 (Figs. 1a, b, S1b, and c), which subsequently stimulated the OSE2 reporter plasmid (Fig. 1c) and upregulated *Spp1* mRNA expression (Fig. 1d) as well as ALP activity (Fig. 1e and f). We also confirmed that lansoprazole induced expressions of *Runx2* mRNA and Runx2 protein in mouse osteoprogenitor cells (Fig. 2a and c) and human osteoblastic cells (Fig. 2b and d). In addition, lansoprazole increased expressions of *Runx2* mRNA at all stages of osteogenic differentiation in both human MSCs (Fig. 2e) and rat BMCs (Fig. S1d). We used BMP-2 to differentiate mouse C3H10T1/2 cells into mature osteoblasts. In contrast, we used osteogenic medium to differentiate human and rat primary BMCs, because we hoped to examine the applicability of lansoprazole in clinical settings. In surgical lengthening of limbs, we differentiate MSCs in patient's bone marrow aspirates into osteoblast-like cells *ex vivo* and inject the differentiated cells into the distraction gap to promote bone formation (Kitoh et al., 2004). We thus added lansoprazole to cultured bone marrow cells that were obtained from pediatric patients undergoing acetabular osteotomy after appropriate written informed consents were given, and found that lansoprazole accelerated the matrix calcium deposition in a dose-dependent manner (Fig. 2f). We next examined the effect of lansoprazole on expanded MSCs, and observed more prominent effects (Fig. 2g) rather than those on the immediately differentiated cells (Fig. 2f). As longer time exposure to 40 μM lansoprazole decreased the number of cells and subsequently reduced the matrix calcium deposition (Fig. 2g), we used 20 μM lansoprazole as a default concentration in the following studies.

### 3.2 Lansoprazole Accelerates Fracture Repair and Osteoblastic Differentiation in a Rat Fracture Model

**Supplemental Fig. 2:**
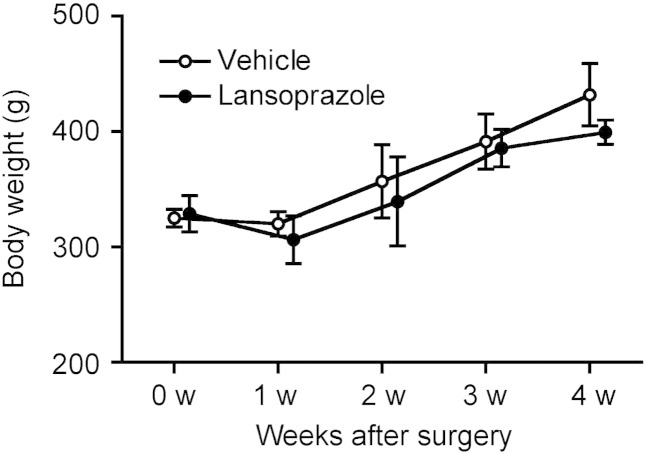
Changes in body weights of vehicle- and lansoprazole-administered rats in a rat model of femoral fracture (*n* = 8 per group). No significance difference was observed between two groups by two-way repeated measures ANOVA.

**Supplemental Fig. 3:**
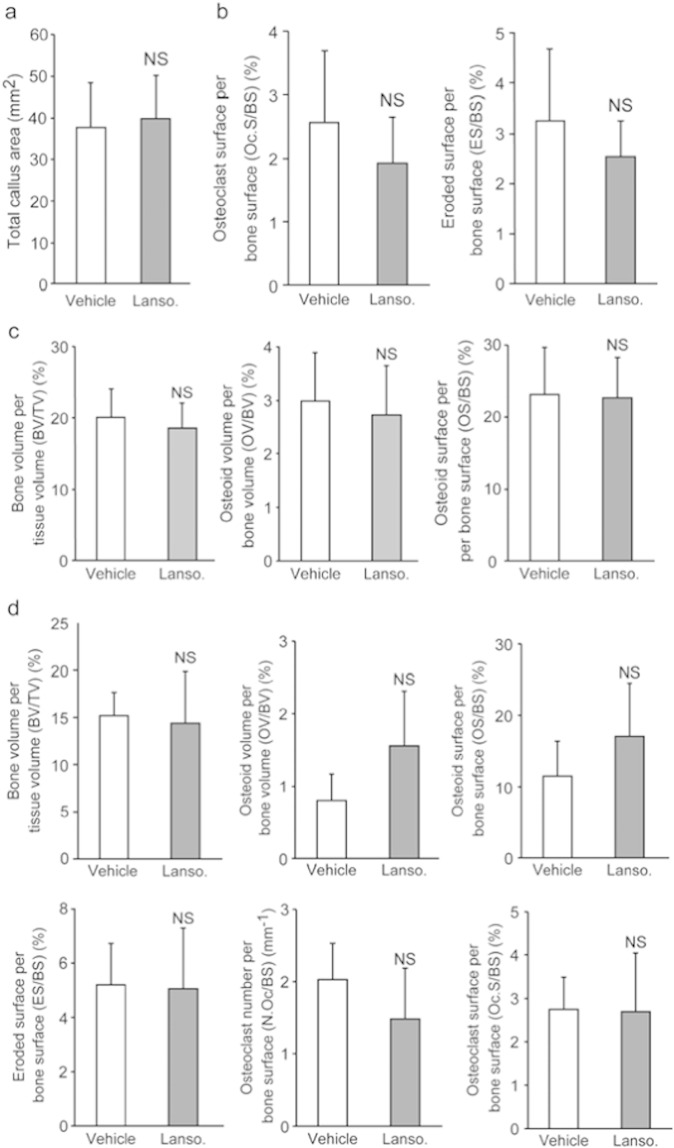
Radiological and histomorphometrical analyses of the fractured and unfractured femurs in vehicle- and lansoprazole-administered rats. (a) Radiological assessment of total callus areas on the anteroposterior and lateral radiographs after a 4-week administration of lansoprazole. (b) Osteoclastic parameters at the injured metaphyses of the fractured femurs after a 4-week administration of lansoprazole. (c) Osteoblastic parameters at the intact metaphyses of the unfractured femurs after a 4-week administration of lansoprazole. (d) Histomorphometric parameters at the intact metaphyses of the unfractured femurs after a 12-week administration of lansoprazole. In (a–d), lanso., lansoprazole; and NS, not significant.

**Supplemental Fig. 4:**
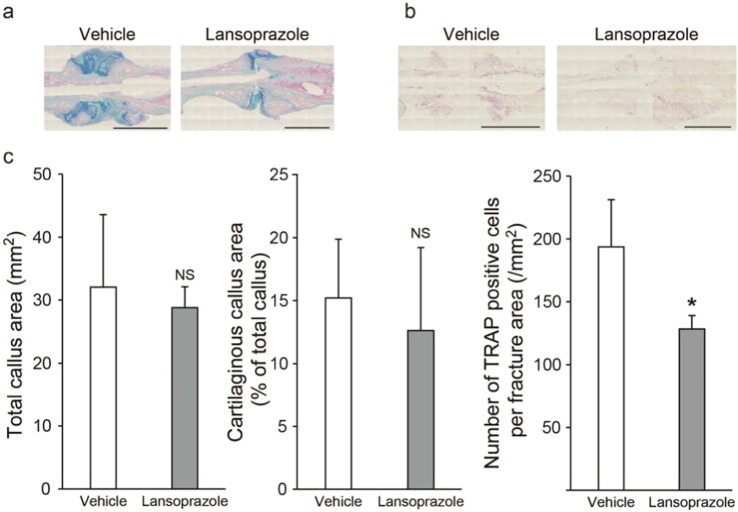
Effects of lansoprazole on chondrogenesis and osteoclastogenesis during an early phase of fracture healing in a rat model of femoral fracture. (a) Representative images of cartilaginous callus formation at the fracture sites detected by alcian blue staining at 2 weeks after fracture (*n* = 4 per group). Dark blue signals show cartilage tissues. Scale bar, 5 mm. (b) Representative images of osteoclast formation at the fracture sites detected by TRAP staining at 2 weeks after fracture (*n* = 4 per group). Red signals show TRAP-positive cells. Scale bar, 5 mm. (a) and (b) are serial sections. (c) Morphometric analysis of (a) and (b). Total callus area was defined as the sum of bridging callus and the anchoring callus. Cartilaginous callus area was normalized to total callus area. The number of TRAP-positive cells was normalized to the fracture area, which was defined as the sum of the total callus area and the underlying cortical bone area. NS, not significant. *p < 0.02 by unpaired *t*-test.

**Fig. 3:**
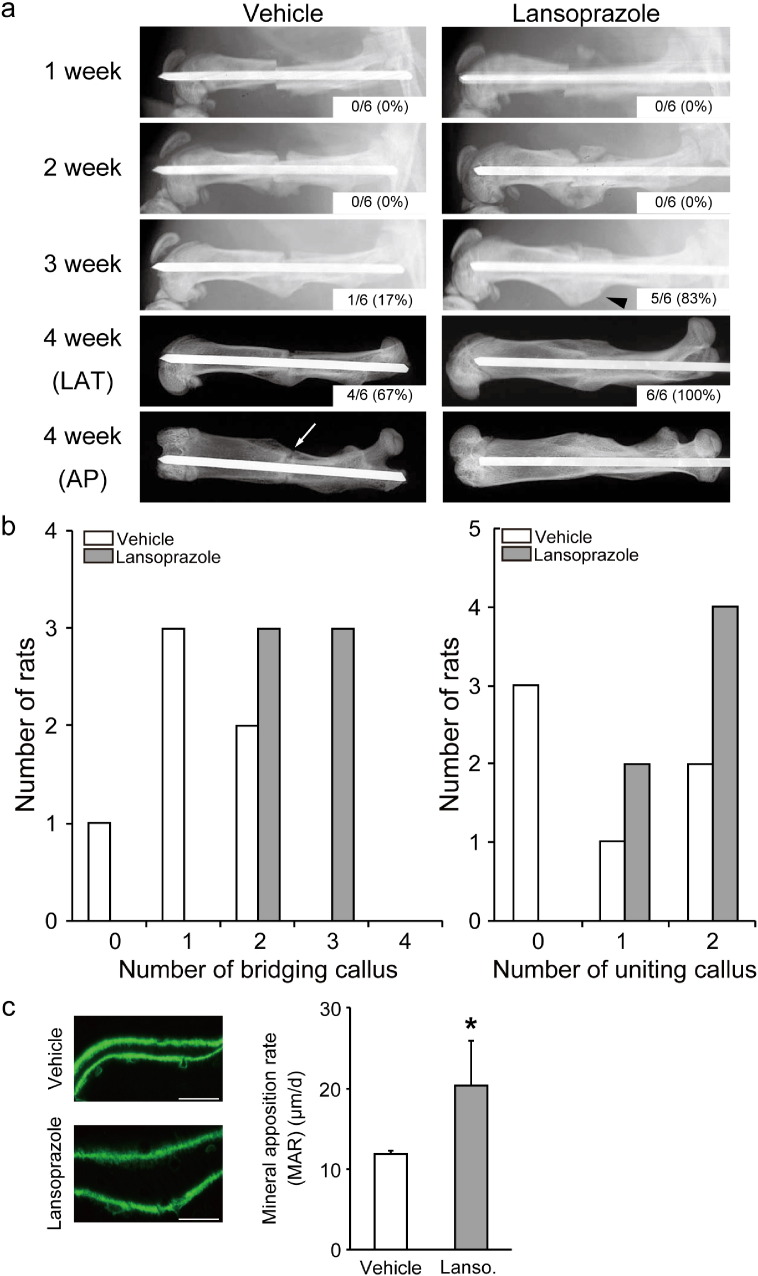
Orally administered lansoprazole accelerates fracture healing in a rat model of femoral fracture. (a) Representative radiographs of vehicle- and lansoprazole-administered rats at 1, 2, 3, and 4 weeks after fracture. Formation of seamless bridging callus (black arrowhead) and incomplete formation of uniting callus (white arrow) are shown. The proportion of rats with the fracture union is indicated in the inset. LAT, lateral view, AP, anteroposterior view. (b) The number of rats showing the indicated number of seamless bridging calluses (left) and complete uniting calluses (right) (*n* = 6 per group). Bridging callus formation on 4 cortices was evaluated on the anteroposterior and lateral radiographs, and the number of calluses was counted (0 to 4). Uniting callus formation was defined by the disappearance of fracture line on the anteroposterior and lateral radiographs, and the number of calluses was counted (0 to 2). (c) Representative images of new bone formation within the anchoring callus assessed by double calcein labeling at 4 weeks after fracture. *p < 0.03 by unpaired *t*-test.

**Fig. 4:**
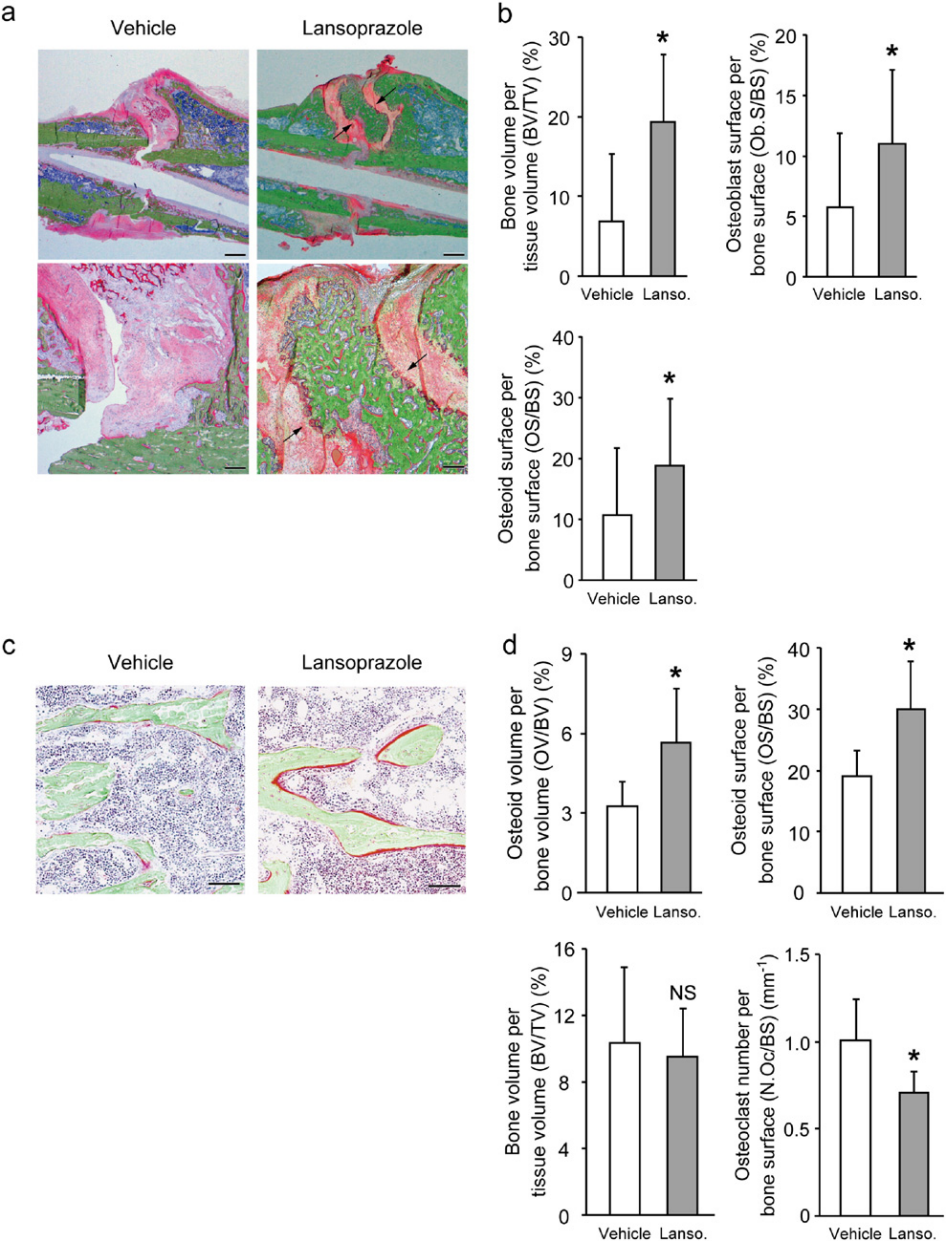
Orally administered lansoprazole accelerated osteoblastic differentiation in a rat model of femoral fracture. (a) Undecalcified bone histology of the fracture sites in vehicle- and lansoprazole-administered rats (Villanueva Goldner staining) 4 weeks after fracture. Green signals show calcified bones. Red signals show granulation tissues spanning edges of osteotomy. Note newly formed bone (arrows) in the lansoprazole-treated rat. Scale bar, 1 mm (upper) and 200 μm (lower). (b) Histomorphometric analyses of undecalcified bone histology of the fracture sites showing enhanced bone formation in lansoprazole-treated rat femurs. The mean ± SD (*n* = 8) are indicated. *p < 0.02 (BV/TV), *p < 0.04 (Ob.S/BS), and *p < 0.05 (OS/BS) by unpaired *t*-test. Lanso., lansoprazole. (c) Undecalcified bone histology of the metaphyses of the fracture femur in vehicle- and lansoprazole-administered rats (Villanueva Goldner stain) 4 weeks after fracture. Red signals show osteoid bones. Note increased osteoid formation in the lansoprazole-treated rat. Scale bar, 100 μm. (d) Histomorphometric analyses of undecalcified bone histology of the metaphyses of the fractured femur. The mean ± SD (*n* = 8) are indicated. *p < 0.04 (OV/BV), *p < 0.02 (OS/BS) and *p < 0.05 (N.Oc/BS) by unpaired *t*-test. Lanso., lansoprazole; and NS, not significant.

To address the clinical relevance of lansoprazole for bone regeneration, we examined the effect of lansoprazole on bone regeneration in a rat model of femoral fracture (Mills and Simpson, 2012). The rats took lansoprazole in drinking water for 4 weeks, which was about 10 times higher than the regular dose used for gastroduodenal ulcer in human. A human equivalent dose (HED) for rodents is 6 to 13 times higher than the human dose (Reagan-Shaw et al., 2008), and the HED of 10 is frequently applied to rodents (Dourson et al., 1996, Foronda et al., 2007, Bian et al., 2009). No significant differences in body weights were observed between both groups at baseline and during the experimental period (Fig. S2). At 3 weeks after fracture, 5 of the 6 rats (83%) achieved fracture union in the lansoprazole-treated group, whereas only 1 of the 6 rats (17%) achieved fracture union in the vehicle-treated group. At 4 weeks, fracture union was observed in all of the lansoprazole-treated rats, whereas it was observed in 4 rats (67%) in the vehicle group (Fig. 3a). The total callus areas, however, were not significantly different between the 2 groups at 4 weeks (Fig. S3a). The formation of bridging callus and uniting callus, which succeeds the formation of anchoring callus (Marino et al., 1979), tended to be better in the lansoprazole group than the vehicle group (Fig. 3b). Dynamic analysis of bone formation with calcein double-labeling showed that lansoprazole enhanced new osteoblastic bone formation in the anchoring callus (Fig. 3c). Histomorphometric analyses at 4 weeks after fracture demonstrated that compared with vehicle-treated rats (*n* = 8), lansoprazole-treated rats (*n* = 8) significantly increased the ratios of bone volume/tissue volume (BV/TV), osteoid surface/bone surface (OS/BS), and osteoblast surface/bone surface (Ob.S/BS) in the gap of the fracture site (Figs. 4a, b, S3b and Table S1). Similarly, at the metaphysis that was penetrated by a wire, lansoprazole significantly enhanced the ratios of osteoid volume/bone volume (OV/BV) and osteoid surface/bone surface (OS/BS), while the bone volume/tissue volume (BV/TV) remained unchanged. In contrast, osteoclast number/bone surface (N.Oc/BS), an osteoclastic parameter, was significantly decreased at the injured metaphysis (Figs. 4c, d, S3b, and Table S2). In addition, at 2 weeks after fracture, when the formation of cartilaginous callus and its subsequent resorption by osteoclasts take place, systemic administration of lansoprazole significantly inhibited osteoclastogenesis but not chondrogenesis at the fracture site (Fig. S4a–c). Taken together, lansoprazole can facilitate osteoblast differentiation and inhibit osteoclast differentiation around the injured site of the bone such as the anchoring callus and the metaphyses penetrated by a wire.

### 3.3 Lansoprazole Activates the Noncanonical BMP–TAK1–p38 MAPK Signaling

**Fig. 5:**
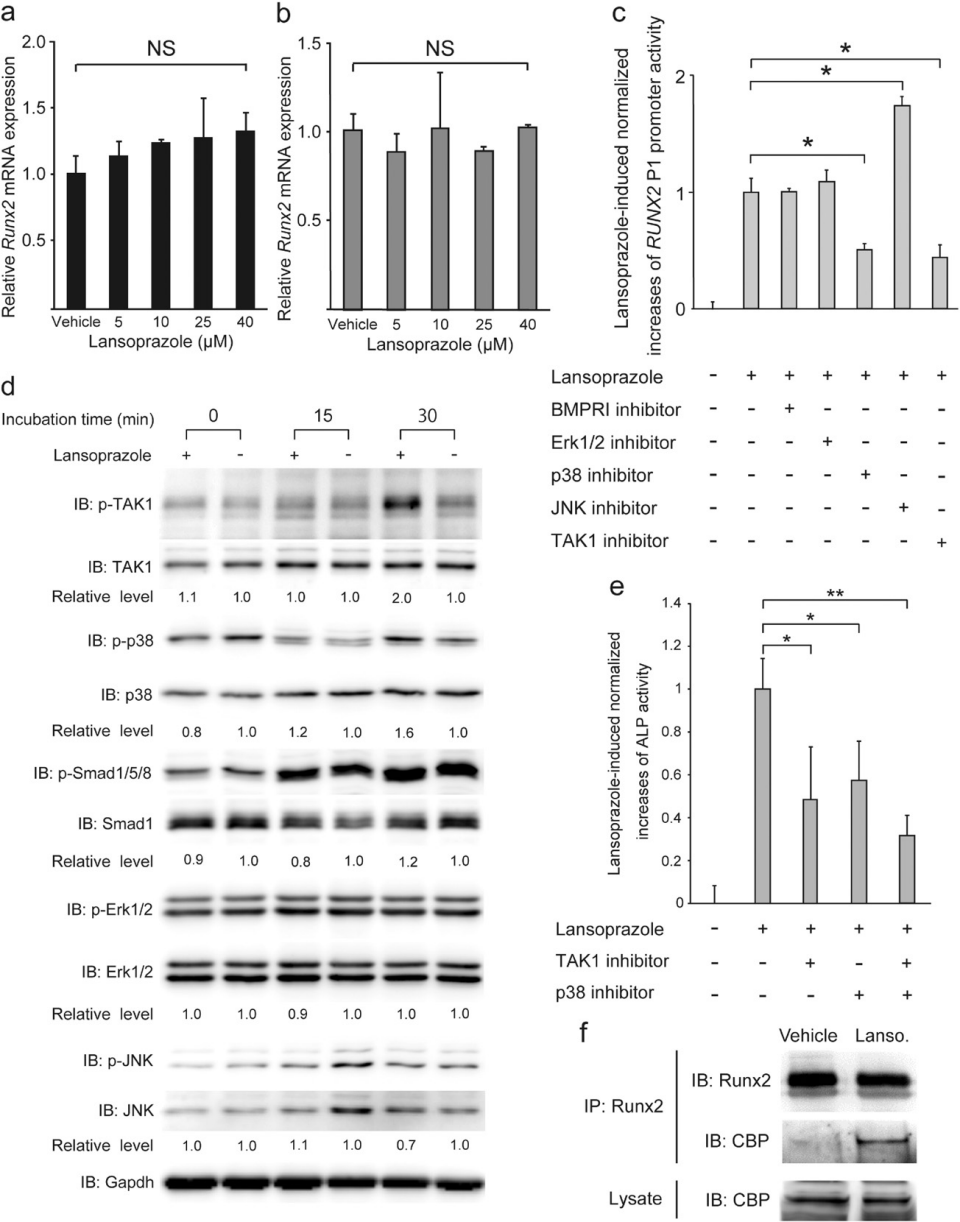
Lansoprazole upregulates the noncanonical BMP–TAK1–p38 MAPK pathway. (a) Expression levels of endogenous *Runx2* mRNA by real-time RT-PCR in native C3H10T1/2 cells without BMP-2 pretreatment. Cells were treated with the indicated concentrations of lansoprazole for 2 d. (b) Expression levels of endogenous *Runx2* mRNA by real-time RT-PCR in native C3H10T1/2 cells pretreated with 400 ng mL^− 1^ of noggin for 2 d. Cells were treated with the indicated concentrations of lansoprazole for 2 d. (c) Effects of BMP signaling-associated protein kinase inhibitors on lansoprazole-induced increases of *RUNX2* P1 promoter activity. C3H10T1/2 cells that stably express pGL4-hRunx2P1-luc2 (C3H10T1/2-hRunx2P1 cells) without BMP-2 pretreatment were treated with the indicated kinase inhibitors for 1 h and subsequently treated with 20 μM lansoprazole for 1 d. In addition, we expected future clinical application of lansoprazole, and we decided to use 20 μM in later analyses. The luciferase activities were normalized to those without lansoprazole and the kinase inhibitors and also to the protein amount of cell lysates. BMPRI kinase inhibitor, dorsomorphin (1 μM); Erk1/2 inhibitor, U0126 (20 μM); p38 inhibitor, SB203580 (20 μM); JNK inhibitor, SP600125 (10 μM); and TAK1 inhibitor, 5Z-7-oxozeaenol (1 μM). *p < 0.0001 by unpaired *t*-test. (d) Phosphorylation of BMP signaling-associated proteins in human MSCs. Relative levels indicate densitometric ratios of phosphorylated to total proteins normalized to the ratio without lansoprazole. (e) Effects of TAK1 and p38 MAPK inhibitors on lansoprazole-induced increases of ALP activity in human MSCs. *p < 0.02 and **p < 0.0003 by unpaired *t*-test. Human MSCs (LONZA) were cultured in the osteogenic medium with or without 20 μM lansoprazole, 100 nM TAK1 inhibitor (5Z-7-oxozeaenol), and 5 μM p38 MAPK inhibitor (SB203580) for 14 d. (f) Lansoprazole activates interactions of Runx2 and CBP. HOS cells were treated with or without 20 μM of lansoprazole for 2 d, and immunoprecipitated with anti-Runx2 antibody. Immunoprecipitated CBP was visualized by immunoblotting. In (a–c), the mean ± SD (*n* = 6) are indicated. NS, not significant by the Jonckheere–Terpstra trend test (a and b).

We next pursued molecular mechanisms of the effects of lansoprazole. We first found that lansoprazole induce expressions of *Runx2* gene even when BMP-2 was not added (Fig. 5a), although the effect was less prominent compared to that observed with BMP-2-added C3H10T1/2 cells (Fig. 2a). Native C3H10T1/2 cells have been shown to produce a small but nonnegligible amount of endogenous BMP-2 (Phimphilai et al., 2006). To confirm that lansoprazole required BMPs, we blocked the binding of BMPs to BMP receptors with noggin, and found that noggin abrogated the effect of lansoprazole (Fig. 5b). Accordingly, lansoprazole cannot substitute for the activity of BMPs to induce osteoblastogenesis in C3H10T1/2 cells, but can enhance the effect of BMPs. We thus scrutinized BMP-signaling pathways leading to Runx2 activation using kinase inhibitors. Inhibitors of p38 MAPK and TAK1 markedly attenuated the effect of lansoprazole, whereas inhibitors of BMP type I receptor (BMPRI) and Erk1/2 had little or no effects (Fig. 5c). Similarly, lansoprazole increased phosphorylation of p38 MAPK and TAK1, while phosphorylation of Smad1/5/8 and Erk1/2 remained unchanged (Fig. 5d). Conversely, inhibitors of p38 MAPK and TAK1 significantly suppressed lansoprazole-induced ALP activity in human MSCs (Fig. 5e). We also confirmed that lansoprazole enhanced the association of Runx2 with a transcriptional coactivator CREB-binding protein (CBP) (Fig. 5f). Taken together, lansoprazole specifically activates the noncanonical BMP–TAK1–p38 MAPK signaling pathway by directly potentiating a molecule downstream of BMPR but upstream of TAK1, and enhances osteoblastic differentiation. Lansoprazole consequently reinforced the ability of Runx2 to recruit a transcriptional activator.

### 3.4 Lansoprazole Activates TRAF6-anchored Polyubiquitination

**Supplemental Fig. 5:**
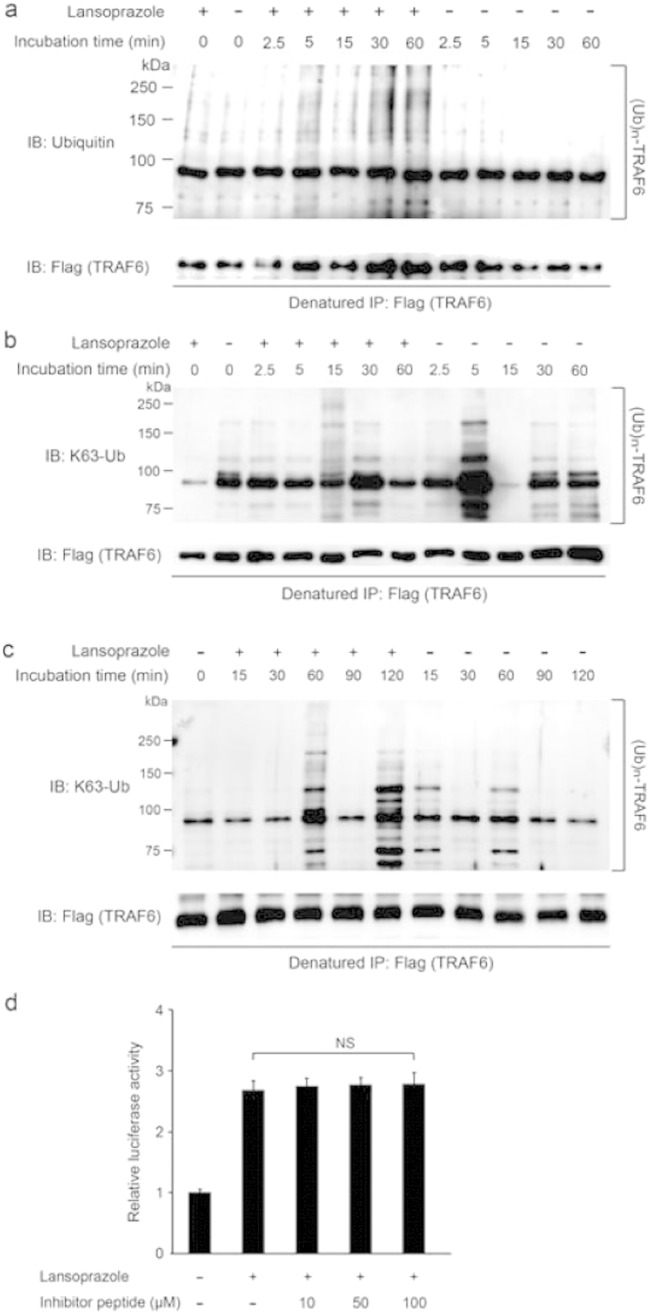
TRAF6-anchored Lys63-linked autopolyubiquitination in cultured cells and effects of TRAF6-inhibitory peptide on lansoprazole-activated human *RUNX2* P1 promoter activity. (a) Lansoprazole induced TRAF6-anchored polyubiquitination with BMP-2 induction in primary mouse calvarial osteoblasts. (b) The immunoprecipitated TRAF6 in Fig. 5c was immunoblotted with an antibody against Lys63-linked polyubiquitin chains. (c) The immunoprecipitated TRAF6 in Fig. 5d was immunoblotted with an antibody against Lys63-linked polyubiquitin chains. (d) The inhibitor peptide that specifically blocks binding of TRAF6 to BMPRI has no effect on lansoprazole-induced activation of *RUNX2* P1 promoter in C3H10T1/2-hRunx2P1 cells. The cells were pretreated with the indicated amounts of the inhibitor peptide for 1 d and subsequently treated with 20 μM lansoprazole for 1 d. Statistical significance is determined by the Jonckheere–Terpstra trend test above the evaluated concentration range of lansoprazole. NS, not significant.

**Fig. 6:**
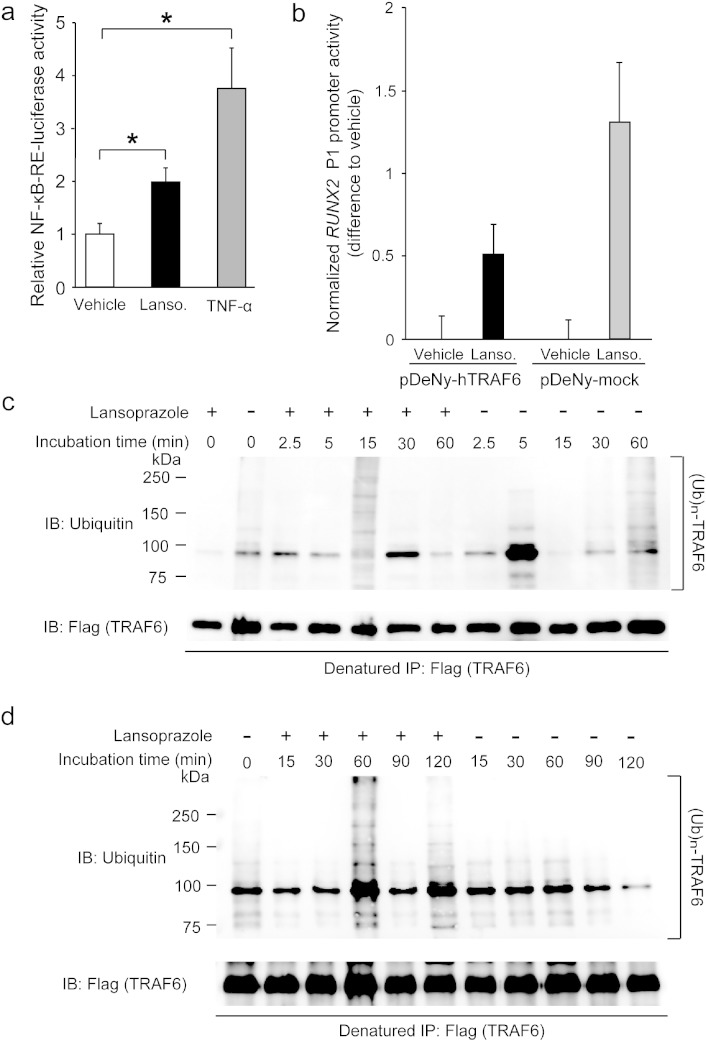
Lansoprazole upregulates TRAF6 polyubiquitination. (a) Activity of NF-κB-responsive-element (RE) luciferase reporter vector in C3H10T1/2 cells. Lanso., lansoprazole (20 μM); TNF-α (1 ng mL^− 1^). *p < 0.0001 by unpaired *t*-test. (b) Effects of a dominant negative TRAF6 (pDeNy-hTRAF6) on a lansoprazole-induced increase of the *RUNX2* P1 promoter activity. A relative luciferase activity was normalized to that of vehicle, and a difference to the vehicle is shown by the mean and SD (*n* = 6). Lanso., lansoprazole. (c) Lansoprazole enhanced TRAF6-anchored polyubiquitination with BMP-2 induction in HEK293 cells. (d) Lansoprazole induced TRAF6-anchored polyubiquitination without BMP-2 induction in HEK293 cells.

We next looked for a target molecule that is activated by lansoprazole and is located downstream of BMPR and upstream of TAK1. An ubiquitin ligase TRAF6 directly binds to the TGF-β–BMP receptors, and is an immediate downstream effector of the receptors (Landstrom, 2010). TRAF6 catalyzes TGF-β–BMP-induced Lys63-linked autopolyubiquitination of TRAF6 itself, which enhances autophosphorylation of TAK1 and subsequently activates NF-κB (Chen, 2005). Unlike Lys48-linked polyubiquitination, which is operational in ubiquitin–proteasome-dependent protein degradation, Lys63-linked polyubiquitination serves to modulate a signaling activity of the polyubiquitination-bearing molecule. We found that lansoprazole increased the NF-κB-responsive luciferase reporter activity (Fig. 6a). Introduction of a dominant-negative TRAF6 inhibited the effect of lansoprazole by 60.8 ((1.308–0.512)/1.308) ± 13.9 (0.182/1.308)% (mean and SD, *n* = 6) (Fig. 6b). As these experiments pointed to the notion that TRAF6 is likely to be a direct target of lansoprazole, we next performed a ubiquitination assay of TRAF6. Upon overexpression of TRAF6 and BMP-2 induction, lansoprazole enhanced TRAF6-anchored polyubiquitination in HEK293 cells and primary osteoblasts (Figs. 6c and S5a). Furthermore, lansoprazole induced TRAF6-anchored polyubiquitination in a serum-free medium and without BMP-2 induction (Fig. 6d), which suggested that TRAF6 was likely to be a direct target molecule activated by lansoprazole. We also found that lansoprazole-mediated TRAF6-anchored polyubiquitination was indeed linked to Lys63 by immunoblotting (Fig. S5b and c).

TRAF6 can bind to BMP type II receptor (BMPRII) in the absence of BMPRI. On the other hand, TRAF6 can bind to BMPRI only when a BMPRI-II complex is formed by BMPs (Yamashita et al., 2008). As lansoprazole enhanced polyubiquitination of TRAF6 even in the absence of BMP ligands, we examined if lansoprazole is able to activate TRAF6 tethered on BMPRII. To this end, we added a TRAF6-inhibitory peptide, which functions as a TRAF6 decoy by binding to the TRAF6-binding motif on BMPRI (Poblenz et al., 2007). As expected, we found that the TRAF6-inhibitory peptide did not compromise lansoprazole-mediated activation of *RUNX2* P1 promoter activity (Fig. S5d). Lansoprazole is thus likely to operate on BMPRII-engaged TRAF6 in the absence of BMP ligands.

### 3.5 Lansoprazole Attenuates Deubiquitination Activity of CYLD *In Vitro*

**Supplemental Fig. 6:**
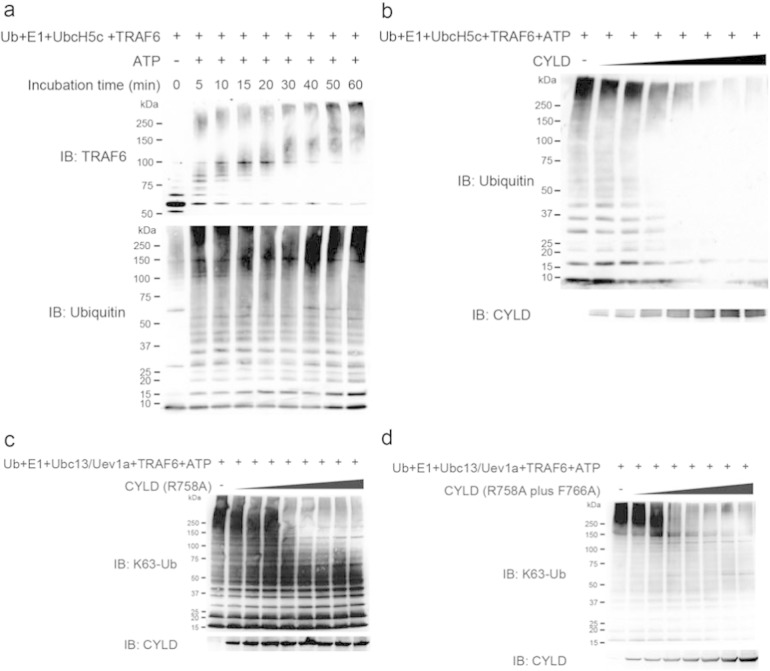
Ubiquitination ligase activity of recombinant TRAF6 and deubiquitination activity of recombinant wild-type and mutant CYLDs. (a) Purified recombinant Flag-tagged TRAF6 catalyzed TRAF6-anchored polyubiquitination. (b) Purified recombinant Flag-tagged wild-type CYLD exerted deubiquitination activity. (c) Purified recombinant Flag-tagged R758A-single-mutant CYLD retained deubiquitination activity. (d) Purified recombinant Flag-tagged R758A and F766A-double-mutant CYLD retained deubiquitination activity.

**Fig. 7:**
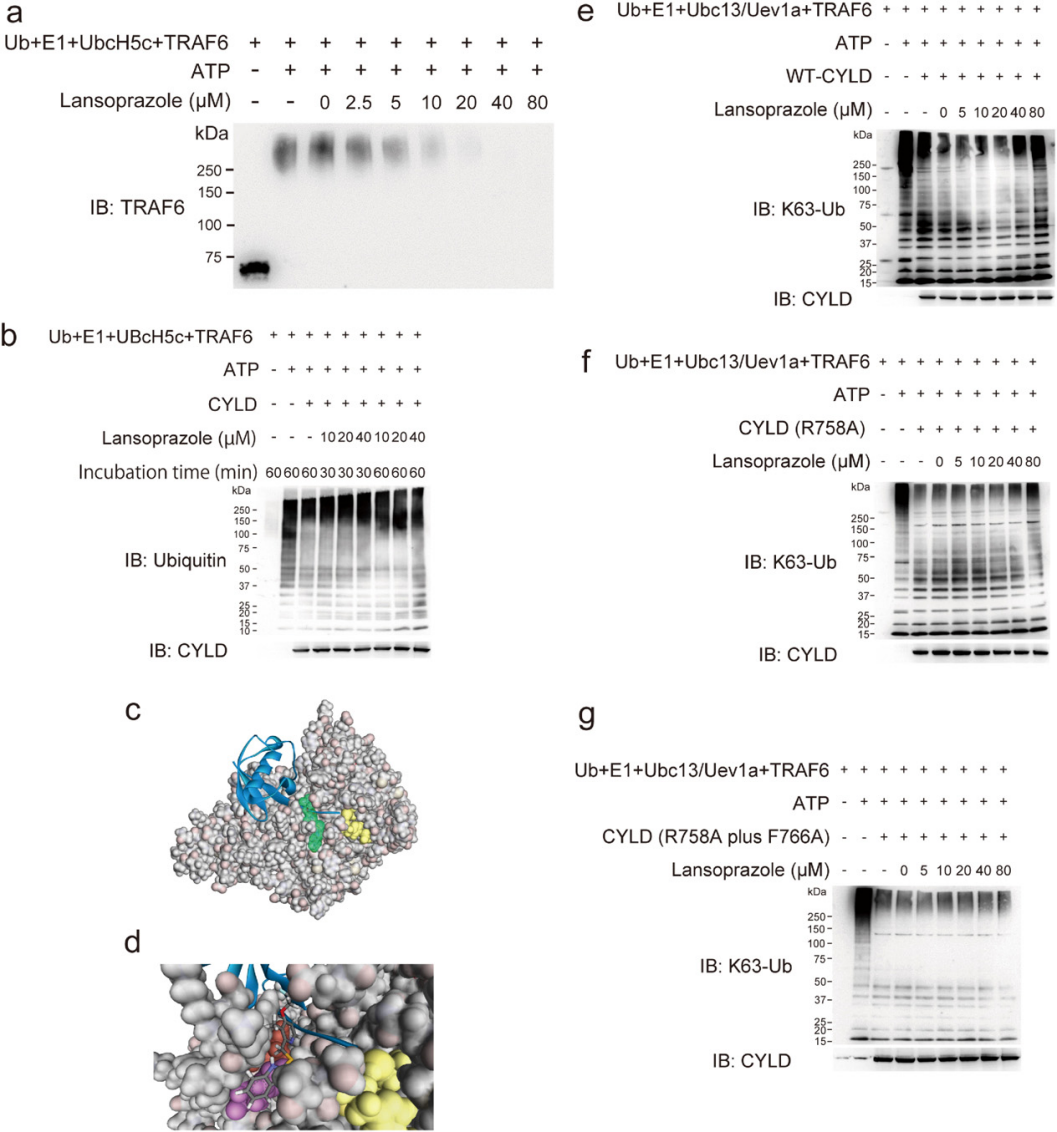
Lansoprazole stably fits in a pocket of CYLD and attenuates its deubiquitination activity *in vitro*. (a) Lansoprazole was incapable of enhancing TRAF6-anchored autopolyubiquitination *in vitro*. (b) Lansoprazole suppressed the deubiquitination activity of CYLD. (c) Simulated crystal structure of CYLD (PDB id: 2VHF) bound to ubiquitin (blue) (PDB id: 1NBF). The C-terminal tail of ubiquitin extends to the active site (yellow). Lansoprazole (green) fits into a pocket to cross the C-terminal tail of ubiquitin. (d) Enlarged image of a pocket where lansoprazole (sticks) fits. Note that lansoprazole crosses the C-terminal tail of ubiquitin (blue). R758 (red) and F766 (purple) are artificially mutated to alanine to deform the lansoprazole pocket. (e) The wild-type CYLD retained the suppressive effects of lansoprazole on CYLD. (f) The R758A-single-mutant CYLD partially abrogated the suppressive effects of lansoprazole on CYLD. (g) The R758A and F766A-double-mutant CYLD completely abolished the suppressive effects of lansoprazole on CYLD.

To prove that the TRAF6 autopolyubiquitination is indeed a target of lansoprazole, we examined the effect of lansoprazole by an *in vitro* ubiquitination assay (Fig. S6a). Unexpectedly, however, lansoprazole failed to enhance the synthesis of TRAF6-anchored polyubiquitination (Fig. 7a). As binding of a small compound to a specific domain of a target molecule mostly inhibits the target molecule rather than activating it, we searched for a molecule that antagonizes TRAF6 polyubiquitination. We found that a deubiquitination enzyme, CYLD, specifically cleaves Lys63-linked polyubiquitin chains, and downregulates TRAF6-mediated signal transduction, which has been characterized in NF-κB activation (Trompouki et al., 2003). As has been previously reported (Xia et al., 2009), CYLD was able to cleave unanchored polyubiquitin chains but not TRAF6-anchored ones *in vitro* (Fig. S6b). We then examined the effect of lansoprazole on CYLD using unanchored polyubiquitin chains, and found that the cleavage was inhibited by pretreatment of lansoprazole in a dose-dependent manner (Fig. 7b).

### 3.6 Lansoprazole Stably Fits in a Pocket of CYLD

**Fig. 8:**
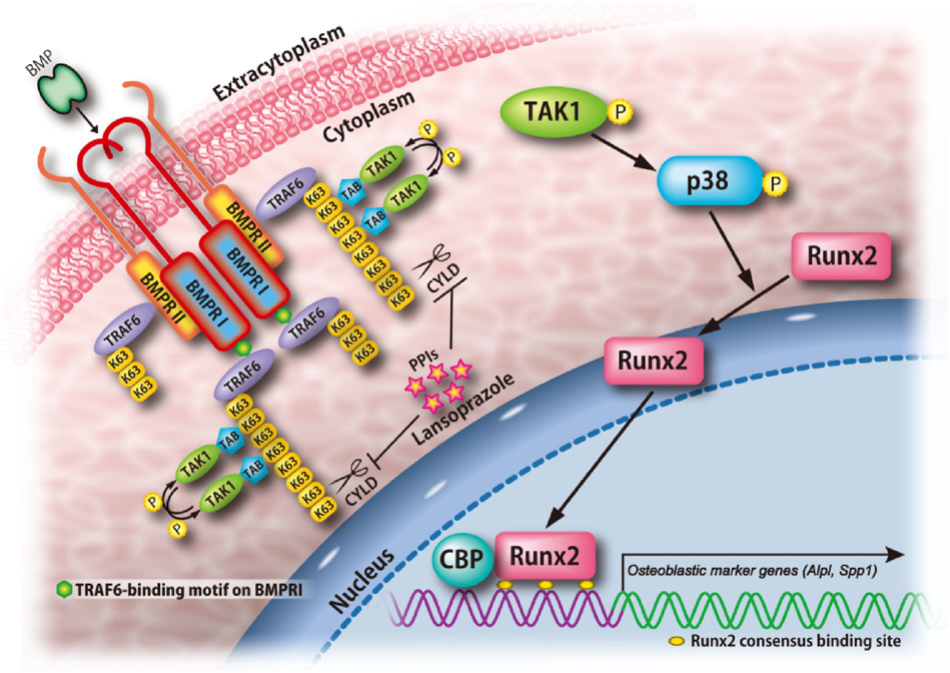
Proposed model of lansoprazole-induced activation of Runx2. Lansoprazole activates TRAF6-catalyzed Lys63-linked autopolyubiquitination of TRAF6 itself by inhibiting a specific deubiquitination enzyme, CYLD. Lys63-linked polyubiquitinated chains of TRAF6, in turn, trigger autophosphorylation of TAK1. Unlike Lys48-linked polyubiquitination in protein degradation, Lys63-linked polyubiquitination of TRAF6 activates TRAF6. The TAK1–p38 MAPK pathway, a noncanonical BMP pathway, then activates transcriptional activity of Runx2, which requires recruitment of a transcriptional coactivator, CBP. The binding of BMP ligand to BMPRI and BMPRII markedly potentiates lansoprazole-induced activation of Runx2.

We next asked if lansoprazole is able to bind to CYLD. An *in silico* search for ligand-binding sites of CYLD disclosed a unique pocket (Fig. 7c). The pocket was located across which the C-terminal tail of ubiquitin was predicted to lie according to structural alignment of CYLD to homologous HAUSP-USP7 that was crystallized with ubiquitin. Optimization of the docking structures predicted that lansoprazole linked to CYLD by hydrogen bond, σ–п conjugation, and п–п interaction (Fig. 7d). The docked structure model suggests that lansoprazole suppresses deubiquitination activity of CYLD by inhibiting the binding of the C-terminal tail of ubiquitin to CYLD. The active site of CYLD is predicted to be located where the C-terminal tail of ubiquitin ends. To prove that lansoprazole binds to the predicted pocket of CYLD, and inhibits its deubiquitination activity, we next employed the Ubc13–Uev1a complex as an E2 enzyme, which specifically catalyzes unanchored Lys63-linked polyubiquitin chains (Xia et al., 2009). We first confirmed that lansoprazole attenuates wild-type CYLD-mediated cleavage of the polyubiquitin chains (Fig. 7e). As simulation of serial alanine substitutions in the identified CYLD pocket predicted that R758 and R766 were essential residues for the binding to lansoprazole, we engineered an R758A-single-mutant CYLD and an R758A and F766A-double-mutant CYLD. Each mutant CYLD retained a dose-dependent deubiquitination activity *in vitro* (Fig. S6c and d), whereas the single mutant CYLD partly and the double mutant CYLD completely abolished lansoprazole-mediated suppression of the deubiquitination activities (Fig. 7f and g). Thus, the mutations exclusively affected responsiveness of lansoprazole but not the deubiquitination activity of CYLD in the absence of lansoprazole. These results indicated that specific binding of lansoprazole to the CYLD pocket prevents the C-terminal tail from reaching the active site, which then facilitates TRAF6-mediated polyubiquitination (Fig. 8).

### 3.7 Lansoprazole Increases Osteoclast Maturation and Bone Resorption Activity

**Fig. 9:**
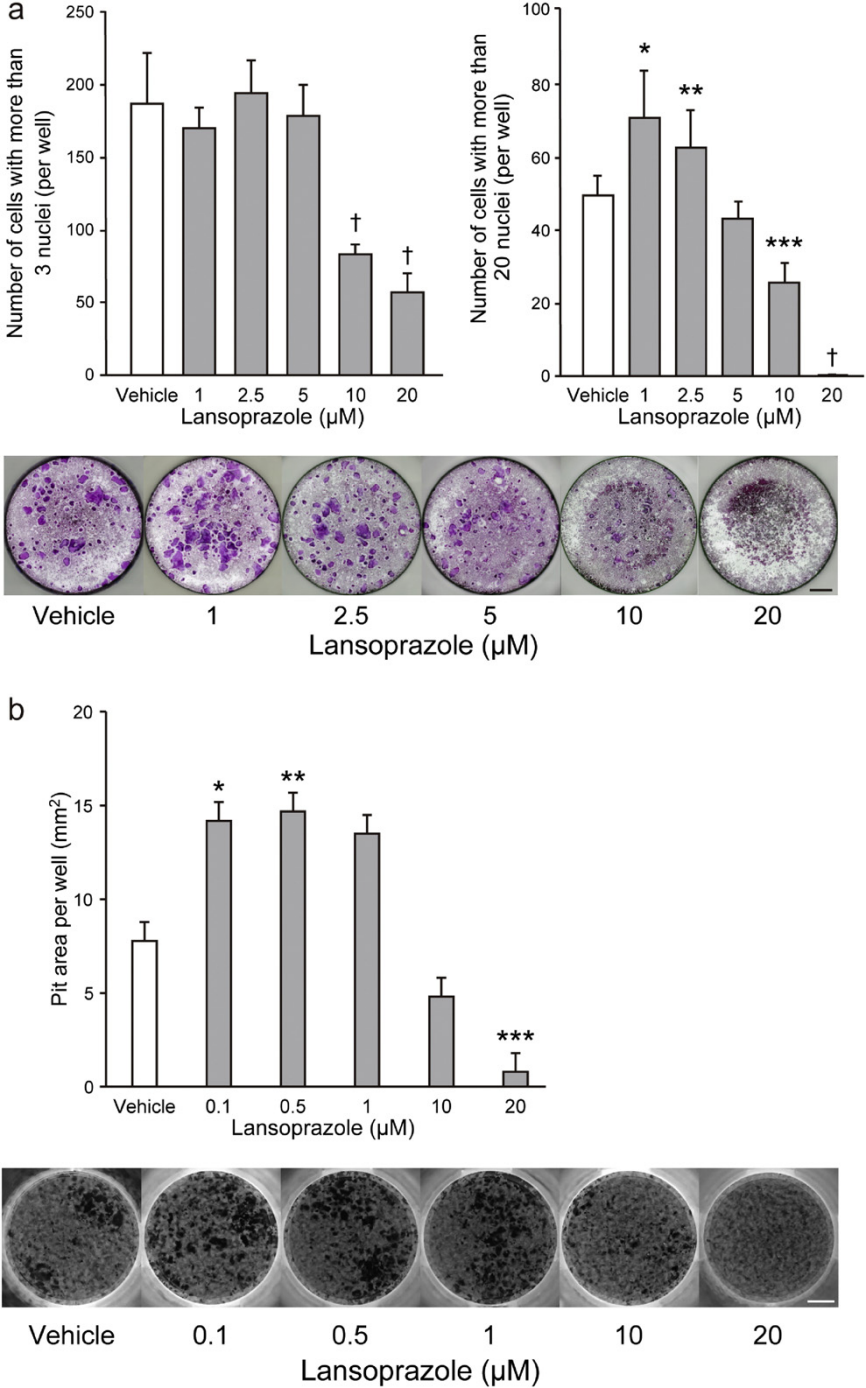
Lansoprazole activates osteoclast formation and bone resorption activity. (a) Effects of lansoprazole on osteoclast formation and maturation. RAW 264.7 cells were cultured for 4 d with or without lansoprazole in the presence of 100 ng mL^− 1^ RANKL. TRAP-positive cells with more than 3 nuclei were counted as osteoclasts. The numbers of total and mature osteoclasts are shown on the top. Representative TRAP-stained cell images are shown at the bottom. †*p* < 0.0000001, *p < 0.0005, **p < 0.04, and ***p < 0.0001 by one-way ANOVA with post-hoc Dunnett analysis. Scale bar, 1 mm. (b) Effects of lansoprazole on osteoclast function. RAW 264.7 were cultured on a calcium phosphate-coated plate with or without lansoprazole in the presence of 100 ng mL^− 1^ RANKL for 7 d. Sizes of the eroded areas are shown on the top. Representative images of the resorption pits are shown at the bottom. Scale bar, 2 mm. *p < 0.04, **p < 0.03, and ***p < 0.02 by one-way ANOVA with post-hoc Dunnett analysis.

To verify whether CYLD is the molecular target of lansoprazole from other aspects, we examined the effects of lansoprazole on osteoclast differentiation and bone-resorbing function using RAW 264.7 cells, in which a critical role of CYLD and TRAF6 has been shown downstream of receptor activator of NF-κB (RANK) — RANKL signaling (Jin et al., 2008, Tanaka et al., 2003). Lansoprazole significantly increased the proportion of large osteoclasts at a concentration of 1 to 2.5 μM compared to vehicle without affecting the total number of osteoclasts (Fig. 9A). On the other hand, higher concentrations of lansoprazole more than 10 μM markedly decreased osteoclasts formation compared to vehicle probably due to cytotoxicity (Fig. 9a). Bone resorption activity, evaluated by the pit formation assay, was significantly increased by addition of lower concentrations of lansoprazole less than 1 μM (Fig. 9b). Lansoprazole is thus capable of enhancing osteoclast maturation and bone resorbing activity *in cellulo*.

### 3.8 Lansoprazole Enhances Osteoclast Differentiation and Induces an Osteomalacia-like Condition *In Vivo*

**Supplemental Fig. 7:**
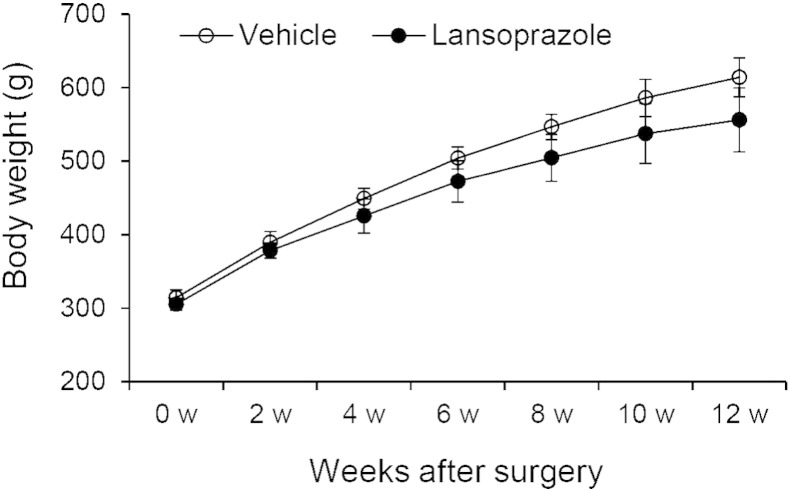
Changes in body weights of unoperated rats, which were administered with either vehicle or lansoprazole for 12 weeks (*n* = 4 per group). No significance difference was observed between two groups by two-way repeated measures ANOVA.

**Fig. 10:**
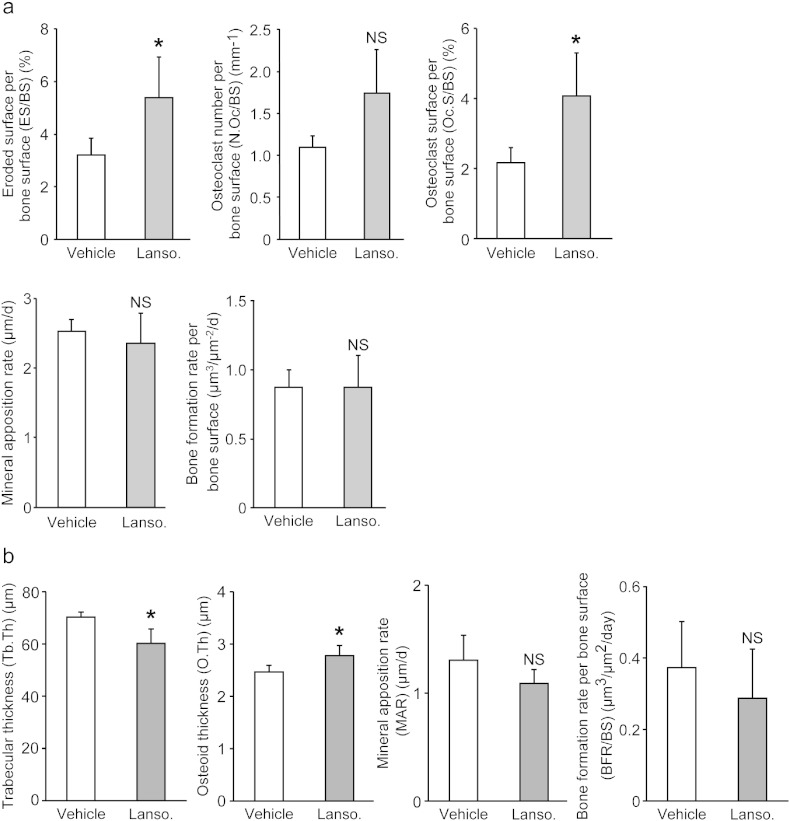
Systemic administration of lansoprazole enhances osteoclastogenesis and induces osteomalacia-like change at the metaphyses of unfractured rat femurs. (a) Histomorphometric analyses of undecalcified histology in rats that were administered with lansoprazole for 4 weeks. The mean ± SD (*n* = 4) are indicated. *p < 0.05 (ES/BS) and *p < 0.03 (Oc.S/BS) by unpaired *t*-test. Lanso., lansoprazole; and NS, not significant. (b) Histomorphometric analyses of undecalcified histology in rats that were administered with lansoprazole for 12 weeks. The mean ± SD (*n* = 4) are indicated. *p < 0.02 (Tb.Th) and *p < 0.04 (O.Th) by unpaired *t*-test. Lanso., lansoprazole; and NS, not significant.

To explore the *in vivo* effect of lansoprazole-mediated enhancement of osteoclast maturation and sustained activation of Runx2, we examined both acute and chronic effects of lansoprazole on physiological cancellous bone remodeling in rats. To this end, rats were fed with the same amount of lansoprazole as that in the fracture model for 4 and 12 weeks, and unfractured femurs were harvested. No significant differences in body weights were observed between both groups at baseline and during the experimental period (Fig. S7). Systemic administration of lansoprazole for 4 weeks significantly elevated osteoclastic parameters, whereas static and dynamic osteoblastic parameters remained unchanged (Figs. 10a and S3c). In contrast, administration lansoprazole for 12 weeks induced an osteomalacia-like condition such as decreased trabecular thickness and increased osteoid thickness in the absence of enhanced osteoclastogenesis (Figs. 10b and S3d).

## 4 Discussion

Our drug repositioning study revealed that lansoprazole, a proton pump inhibitor, has bone anabolic activity through upregulation of Runx2 and serves as an enhancer of BMP signaling. Using the drug repositioning strategy, Mundy and colleagues identified that statins that are commonly prescribed for hyperlipidemia enhance the *BMP2* promoter activity (Mundy et al., 1999). Similar to lansoprazole, statins stimulate bone formation in animal models (Gutierrez et al., 2008). However, statins may (Edwards et al., 2000) or may not (Esposito et al., 2013) increase bone mineral density and prevent osteoporotic fractures in postmenopausal women. BMP-2 is a potent osteoinductive agent that is required for the initiation of fracture healing (Tsuji et al., 2006) and for the development and maturation of osteoblasts (Chen et al., 2012a). BMP-2, however, is also capable of inducing pluripotent mesenchymal cells into multiple lineages such as osteoblasts, chondrocytes, and adipocytes (Date et al., 2004). Controversial effects on bone metabolism of statins may be attributed to the diverse effects of BMP-2. On the contrary, Runx2 exclusively induces osteochondroblastogenesis (Komori, 2010), but Runx2 alone is not sufficient to induce the maturation of osteoblasts (Lee et al., 2000). Runx2 is upregulated by BMPs (Phimphilai et al., 2006), and the two molecules orchestrate to induce the development and maturation of osteoblasts (Leboy, 2006). Upregulation of BMPs by statins and upregulation of Runx2 by lansoprazole are expected to coordinately accelerate osteoblastogenesis, but further studies are required.

BMP ligands bind to BMPRII, and facilitates the formation of a heterotetrameric receptor complex comprised of BMPRI and BMRPII, which then activates the canonical pathway through phosphorylation of the receptor-regulated Smads as well as the noncanonical MAPK pathways (Katagiri and Tsukamoto, 2013). For Runx2 activation, both canonical and noncanonical pathways play essential roles in mesenchymal precursor cells (Lee et al., 2002). Among the noncanonical pathways, activation of the TAK1–p38 MAPK axis is essential for Runx2 activation in mesenchymal precursor cells (Greenblatt et al., 2010). BMP-mediated formation of a BMPRI-II complex additionally induces accumulation of TRAF6 on the complex. TRAF6 is a ubiquitin ligase as well as a substrate polyubiquitinated by itself. The accumulation of TRAF6 enhances the synthesis of Lys63-linked autopolyubiquitin chains. The polyubiquitin chains confer a scaffold for the TAB-mediated adjacent placement of two TAK1 molecules, which leads to autophosphorylation of TAK1 (Xia et al., 2009, Landstrom, 2010, Sorrentino et al., 2008). The phosphorylated TAK1 then phosphorylates p38 MAPK. The activated p38 MAPK also activates other transcriptional factors that are essential for bone formation including Osterix and distal-less homeobox 5 (Dlx5) (Ulsamer et al., 2008, Ortuno et al., 2010). Dissection of underlying molecular mechanisms of the effect of lansoprazole revealed that lansoprazole has little or no effects on the canonical pathway, but activates the noncanonical TRAF6–TAK1–p38 MAPK pathway.

The tumor suppressor CYLD specifically digests Lys63-linked polyubiquitin chains that are attached to the TNF receptor-associated factors, TRAF2 and TRAF6, as well as an essential NF-κB signaling component, NF-κB essential modulator (NEMO) (Brummelkamp et al., 2003, Kovalenko et al., 2003, Trompouki et al., 2003). Among the CYLD-catalyzed molecules, TRAF6 is the only molecule in the TGF-β–BMP pathway (Xie, 2013). In previous *in vitro* studies using purified proteins, CYLD was able to cleave unanchored polyubiquitin chains, but not polyubiquitin chains conjugated to TRAF6 (Xia et al., 2009). An ubiquitin-conjugating E2 enzyme, UbcH5c, catalyzes both Lys63- and Lys48-polyubiquitin chains either anchored or unanchored to TRAF6, whereas another E2 enzyme, Ubc13, efficiently catalyzes unanchored Lys63-polyubiquitin chains (Xia et al., 2009). We thus examined the effect of lansoprazole on the deubiquitination activity of CYLD using unanchored polyubiquitin chains synthesized with UbcH5c (Fig. 7b) and Ubc13 *in vitro* (Fig. 7e), and we only used Ubc13 for the subsequent mutagenesis studies of CYLD (Fig. 7f and g). It is interesting to note that even TRAF6-catalyzed unanchored polyubiquitin chains are capable of activating TAK1 (Xia et al., 2009). Thus, in addition to the induction and acceleration of TRAF6-anchored polyubiquitin chains by lansoprazole (Fig. 6c and d), stabilization of unanchored polyubiquitin chains by lansoprazole (Fig. 7b and e) may partly account for the activation of TAK1.

TRAF6 is an essential factor for osteoclastogenesis as evidenced by osteopetrotic phenotypes in TRAF6-deficient mice (Lomaga et al., 1999, Naito et al., 1999). TRAF6 is indispensable for signal transduction downstream of RANK–RANKL pathway by binding to the cytoplasmic tail of RANK (Inoue et al., 2000). Dissection of TRAF6 domains demonstrated that different domains of TRAF6 regulate osteoclast differentiation, maturation, and function (Kobayashi et al., 2001). The RING finger domain of TRAF6, which is critical for the ubiquitin E3 ligase activity, is not crucial for osteoclast differentiation but is essential for full maturation and activation of osteoclasts. Likewise, CYLD-deficient mice, in which more polyubiquitinated TRAF6 was accumulated, had enlarged osteoclasts (Jin et al., 2008). In addition, bone marrow cells from the CYLD-deficient mice exhibited enhanced expressions of osteoclastic marker genes upon RANKL stimulation. Therefore, lansoprazole-mediated increased osteoclast maturation and bone resorbing activity *in cellulo*, which were also demonstrated at the intact metaphyses of the unfractured femurs *in vivo*, provide supportive evidence that CYLD and TRAF6 are the molecular targets of lansoprazole. In contrast, in a rat model of femoral fracture, lansoprazole promotes osteoblastic maturation and impairs osteoclastogenesis both at the fracture site and the injured metaphyses, where BMP is induced for bone regeneration. Although the mechanisms underlying the different effects of lansoprazole on osteoclastogenesis between fractured and unfractured bones remain unknown, suppression of osteoclastogenesis at the fracture site by lansoprazole is predicted to be favorable for facture healing.

The FDA announced a safety alert regarding the possible association between the risk of fractures and the use of PPIs in aged individuals (Ngamruengphong et al., 2011), although the exact mechanisms remain controversial. The adverse effect may be attributed to decreased absorption of insoluble calcium due to the elevated gastric pH observed in aged women taking PPIs (O'Connell et al., 2005). Along with this observation, mice deficient for *Cckbr* encoding a gastrin receptor that facilitates gastric acid secretion have modest hypocalcemia (Schinke et al., 2009), and mice deficient for gastric H^+^–K^+^ ATPase beta-subunit also show decreased bone mineral density (Fossmark et al., 2012). A retrospective study, however, shows that patients taking high doses of PPIs do not have low bone mineral density or increased rate of bone loss (Targownik et al., 2010). We demonstrated that lansoprazole could induce osteopenia and fragility at the metaphyses of unfractured long bones by alteration of the trabecular structures to an osteomalacia-like condition and increased bone resorption of the trabeculae. Previously, the maturational blockage of osteoblasts and osteopenia have been reported in mice overexpressing Runx2 (Liu et al., 2001). PPIs-mediated increased chance of fragility fractures is thus likely to be attributed to sustained activation of Runx2 and partly to enhancement of osteoclast maturation.

Lansoprazole and other PPIs have been widely used for more than 20 years in the prevention and treatment of the acid-related disorders such as gastroduodenal ulcers and reflux esophagitis, although the molecular bases of the proton pump-inhibiting effects of lansoprazole remains elusive. In spite of their efficacy, long-term use or high dose of PPIs have been implicated to provoke several adverse effects including increased pneumonia, *Clostridium difficile*-associated disease, fragility-related fractures, neoplasms, iron deficiency, and acute interstitial nephritis (Moayyedi and Leontiadis, 2012, Chen et al., 2012b). As chronic activation of TGF-β–BMP signal transmission causes detrimental cellular processes including fibrosis, atherosclerosis, tumorigenesis, cancer metastasis, and autoimmune disorders (Akhurst and Hata, 2012), enhancement of the TGF-β–BMP signaling by lansoprazole may partly account for some of these adverse effects. A recent report showed that osteogenic induction of human MSCs in the presence of lansoprazole (10–1000 μM) for a longer period (> 14 d) decreased ALP activity (Costa-Rodrigues et al., 2013). In addition, higher concentrations (100–1000 μM) of lansoprazole for 7 d or more attenuated cell proliferation by inducing apoptosis. In contrast, we confirmed increased ALP activity in human bone marrow-derived mesenchymal cells that were treated with 5–40 μM lansoprazole for up to 6 d (Fig. 1e and f). Therefore, even in fresh bone marrow cells, longer exposure of lansoprazole may inhibit ALP activity at lower concentrations. An off-label higher dose of lansoprazole in the context of osteogenesis promotion is thus expected to be limited to either short-term or local usage.

We have started developing a biodegradable artificial bone that enables a sustained release of concentrated lansoprazole. We believe that the combined use of lansoprazole with biomaterials would lead to the invention of a unique bone graft substitute that possesses the capacity of osteoinduction in addition to osteoconduction. In addition, lansoprazole along with the osteogenic medium may be able to enhance osteoblastic differentiation of bone marrow derived mesenchymal stromal cells *ex vivo*. Although detailed absorption, distribution, metabolism, excretion, and toxicity (ADMET) analysis is required for *ex vivo* application of lansoprazole, the probability of unpredicted adverse effects is expected to be lower than chemical compounds that have never been used in humans.

The following are the supplementary data related to this article.

Supplementary material 1.

Supplementary material 2.

## Author Contributions

Study design: KM, HK, and KO. Data collection: KM and BO. Structural modeling: TO. Data analysis and interpretation: KM, BO, MI, and AM. Drafting manuscript: KM, HK, and KO. NI and KO take responsibility for the integrity of the data analysis.

## Conflict of Interest

The authors have declared that no competing interests exist.
