# Supplementary material for: Lansoprazole Upregulates Polyubiquitination of the TNF Receptor-Associated Factor 6 and Facilitates Runx2-mediated Osteoblastogenesis
Source: eBioMedicine. 2015 Nov 17;2(12):2046–61. doi: 10.1016/j.ebiom.2015.11.024 (PMC4703748; doi:10.1016/j.ebiom.2015.11.024)
Supplement: Supplementary file 1 — Supplementary material 1. [file mmc1.pdf]

Supplemental Materials and Methods

Cell culture

We purchased human MSCs from LONZA, and grown them according to the manufacturer's instructions. We serially subcultured MSCs and mesenchymal progenitors 2 to 3 times to confluency. Lansoprazole was added in either passage 0, 1, 2, or 3. Cells were lysed in 1% Triton X-100, and ALP activity of cell lysate was determined by the colorimetric analysis using p-nitrophenylphosphate (Wako Pure Chemical Ind.). The activity was normalized to the protein amount of cell lysates. For ALP staining, cells were fixed with 10% neutral formalin and stained with BCIP-NBT solution (GE Healthcare). For detecting matrix calcium deposition, osteogenically induced cells were fixed with 95% ethanol and stained with 0.1% alizarin red solution (Sigma-Aldrich). Cells were imaged at room temperature with a DSC-WX300 camera (Sony). Images were processed and analyzed with Photoshop CS6 (Adobe).

Isolation of primary osteoblasts

Primary osteoblasts were isolated from 12 calvariae of neonatal (2–3 days old) mice. Calvariae were digested for 10 minutes at 37°C in an enzyme solution containing 0.5% collagenase (Worthington) and 0.2% dispase (Life Technologies) in α-MEM. Calvariae were digested a total of 5 times, and the cells extracted after the last 4 digestions were combined and cultured in α-MEM containing 15% FBS. The cells were transfected with a cDNA clone by electroporation using the NEPA21 electro-kinetic transfection system (Nepa Gene).

Screening of 1,186 FDA-approved drugs

We constructed the reporter plasmid, pGL4-hRunx2P1-luc2, by inserting the human *RUNX2* P1 promoter region (-1500 to +468) into pGL4.10 (Promega). We then generated a stable C3H10T1/2 cell line that expressed pGL4-hRunx2P1-luc2 (C3H10T1/2-hRunx2P1 cells) using G418. We confirmed that human recombinant BMP-2 increased the luciferase activity ~2-fold. For screening, the stable cells seeded onto 96-well plates were grown in medium supplemented with 10 ng mL^{-1} recombinant BMP-2 (PeproTech) for 1 d. We then added each of 1186 chemical compounds (Prestwick Chemical Library, Prestwick chemical) at a final concentration of 10 μM, and harvested cells in Passive Lysis Buffer (Promega) after incubating for 1 d. The concentration of 10 μM was recommended by the manufacture and has been successfully used in our previous studies (Bian et al., 2009; Matsushita et al., 2013; Phimphilai et al., 2006; Takamatsu et al., 2014; Yamamoto et al., 2013). We excluded 14 opioids from the library due to the import embargo. We determined luciferase activity by Luciferase Assay System (Promega) and PowerScan 4 (DS Pharma Biomedical) according to the manufacturer's instructions. We arbitrarily set a cutoff value of relative luciferase activity to 1.5 in triplicated experiments and repeated the luciferase assay six more times,

which narrowed down to a candidate compound, lansoprazole. Lansoprazole (10 μM) increased the *RUNX2* P1 promoter-driven firefly luciferase activity by a factor of 2.00 ± 0.23 (n = 18) compared to vehicle.

Quantitative RT-PCR analysis

Total RNA was extracted using RNeasy Micro kit (Qiagen) and reverse-transcribed with ReverTraAce (Toyobo) using oligo-dT primers (Invitrogen). We used LightCycler 480 System (Roche) with SYBR Premix Ex Taq II (Takara Bio) for all real-time RT-PCR experiments.

Reagents

Lansoprazole was purchased from Sigma-Aldrich. Recombinant human BMP-2 and Noggin were obtained from PeproTech. The following kinase inhibitors were used: 5Z-7-oxozeaenol (Calbiochem-Merck Millipore), SP600125 and SB203580 (Wako Pure Chemical Ind.), U0126 (Cell Signaling Technology), and dorsomorphin (Sigma-Aldrich). TRAF6 Inhibitory Peptide Set was purchased from Imgenex.

Western blot analysis and immunoprecipitation

Blotted molecules were probed with the following antibodies: anti-Runx2/Cbfa1 from MBL; anti-Smad1, anti-phospho-Smad1/5/8, anti-p44/p42 MAPK, anti-SAPK/JNK, anti-p38 MAPK, anti-phospho-p44/p42 MAPK, anti-phospho-SAPK/JNK, anti-phospho-p38 MAPK, anti-phospho-TAK1, anti-TRAF6, anti-Ub (P4D1), and anti-U2AF35 from Santa Cruz Biotechnology; anti-GAPDH from Sigma-Aldrich; anti-Ub-K63 from eBioscience; anti-TAK1 from Upstate; anti-CYLD from Cell Signaling Technology; and anti-KAT3A/CBP antibody from Abcam. The band intensities were quantified using ImageQuant TL (GE Life Sciences).

Plasmids and transfection

The 6×OSE2-luc+ reporter vector was a kind gift of Dr. Toshihisa Komori (Nagasaki University). Runx2 cDNA clone was purchased from OriGene Technologies. Luciferase reporter vector containing tandem repeats of a NF-κB-responsive element was previously described (Matsuguchi et al., 2000). C3H10T1/2 cells in a 10-cm dish were transiently transfected with FuGene 6 (Promega) or X-tremeGENE 9 (Roche) following the manufacturer's instructions. After transfection, cells were split in a 96-well plate and subjected to further experiments. Firefly luciferase activities were normalized to Renilla luciferase activities originating from co-transfected phRL-TK vector (Promega). An expression vector for dominant negative human TRAF6 cDNA (pDeNy-hTRAF6) was purchased from InvivoGen. We constructed pDeNy-mock by eliminating hTRAF6 cDNA from pDeNy-hTRAF6. C3H10T1/2-hRunx2P1 cells without BMP-2 pretreatment

were transiently co-transfected with pDeNy-hTRAF6 or pDeNy-mock and pSV-β-Galactosidase Control Vector (Promega) in a 10-cm dish. After 2 d, cells were split into a 96-well plate, and subsequently treated with 20 μM lansoprazole for 1 d. Cells were lysed in Passive Lysis Buffer (Promega), and the luciferase and β-galactosidase activities were determined using Luciferase Assay System and Beta-Glo Assay System (Promega), respectively. Firefly luciferase activities were normalized to β-galactosidase activities and also to the mean of vehicle.

Immunofluorescence analysis

HOS cells grown on coverslips were stimulated with 20 μM lansoprazole for 3 d. Cells were then fixed with 4% paraformaldehyde solution for 30 min and permeabilized with 0.3% TritonX-100 for 10 min. Cells were incubated with anti-Runx2 antibody overnight at 4°C and subsequently with anti-mouse FITC secondary antibody (Vector laboratories). Coverslips were mounted in Vectashield Mounting Medium with DAPI (Vector Laboratories). All histological images were obtained at room temperature using a DP72 camera (Olympus) mounted on an IX71 microscope (Olympus) and LUCPLFL N 20× and 0.45 objective lens (Olympus). Acquired images were processed and analyzed with an imaging software cellSens (Olympus).

Docking simulation

Docking of lansoprazole with CYLD was simulated *in silico*. We searched for ligand-binding sites on the surface of CYLD using MolSite (Fukunishi and Nakamura, 2011), FINDSITE (Brylinski and Skolnick, 2008), and LigSite (Huang and Schroeder, 2006). We docked lansoprazole to the identified pocket using Sievgene (Fukunishi and Nakamura, 2011) and optimized the docked structure using the Energy Minimization module of Discovery Studio 3.5. The complex model of CYLD and ubiquitin was constructed from the apo structure of CYLD and the holo structure of HAUSP-USP7. The structure of CYLD (PDB id: 2VHF) was superimposed on that of HAUSP-USP7 in complex with ubiquitin (PDB id: 1NBF) using structural alignment software MICAN (Minami et al., 2013). Serial alanine substitutions in the identified CYLD pocket were simulated with the Mutation Energy module of Discovery Studio 3.5.

Animal management

All rats were individually housed in a cage with a solid plastic floor. Breeding room conditions were automatically controlled at 22 ± 3°C on a 12 hour light-dark schedule. Rats fed a Good Laboratory Practice-compliant diet with a proper balance of cereals, proteins, and minerals (CLEA Rodent Diet CE-2, CLEA Japan) and drank lansoprazole- or vehicle-containing water *ad libitum*.

Supplemental References

Bian, Y., Masuda, A., Matsuura, T., Ito, M., Okushin, K., Engel, A. G. & Ohno, K. 2009. Tannic acid facilitates expression of the polypyrimidine tract binding protein and alleviates deleterious inclusion of CHRNA1 exon P3A due to an hnRNP H-disrupting mutation in congenital myasthenic syndrome. Human molecular genetics. 18, 1229-37.

Brylinski, M. & Skolnick, J. 2008. A threading-based method (FINDSITE) for ligand-binding site prediction and functional annotation. Proc Natl Acad Sci U S A. 105, 129-34.

Fukunishi, Y. & Nakamura, H. 2011. Prediction of ligand-binding sites of proteins by molecular docking calculation for a random ligand library. Protein Sci. 20, 95-106.

Huang, B. & Schroeder, M. 2006. LIGSITEcsc: predicting ligand binding sites using the Connolly surface and degree of conservation. BMC Struct Biol. 6, 19.

Matsuguchi, T., Musikacharoen, T., Ogawa, T. & Yoshikai, Y. 2000. Gene expressions of Toll-like receptor 2, but not Toll-like receptor 4, is induced by LPS and inflammatory cytokines in mouse macrophages. J Immunol. 165, 5767-72.

Matsushita, M., Kitoh, H., Ohkawara, B., Mishima, K., Kaneko, H., Ito, M., Masuda, A., Ishiguro, N. & Ohno, K. 2013. Meclozine facilitates proliferation and differentiation of chondrocytes by attenuating abnormally activated FGFR3 signaling in achondroplasia. PLoS One. 8, e81569.

Minami, S., Sawada, K. & Chikenji, G. 2013. MICAN: a protein structure alignment algorithm that can handle Multiple-chains, Inverse alignments, C(alpha) only models, Alternative alignments, and Non-sequential alignments. BMC Bioinformatics. 14, 24.

Phimphilai, M., Zhao, Z., Boules, H., Roca, H. & Franceschi, R. T. 2006. BMP signaling is required for RUNX2-dependent induction of the osteoblast phenotype. J Bone Miner Res. 21, 637-46.

Takamatsu, A., Ohkawara, B., Ito, M., Masuda, A., Sakai, T., Ishiguro, N. & Ohno, K. 2014. Verapamil protects against cartilage degradation in osteoarthritis by inhibiting Wnt/beta-catenin signaling. PLoS One. 9, e92699.

Yamamoto, R., Matsushita, M., Kitoh, H., Masuda, A., Ito, M., Katagiri, T., Kawai, T., Ishiguro, N. & Ohno, K. 2013. Clinically applicable antianginal agents suppress osteoblastic transformation of myogenic cells and heterotopic ossifications in mice. Journal of bone and mineral metabolism. 31, 26-33.

Supplemental Table 1. Bone histomorphometry at the fracture sites

	BV/TV, %	Ob.S/BS, %	OS/BS, %
Vehicle	6.9 ± 8.5	5.7 ± 6.1	10.7 ± 11.0
Lansoprazole	19.4 ± 17.3	11.0 ± 7.1	18.9 ± 10.8
Statistical significance	$p < 0.02$	$p < 0.04$	$p < 0.05$

Means ± SD are indicated ($n = 8$ per group).

BV, bone volume; TV, tissue volume; Ob.S, osteoblast surface; BS, bone surface; OS, osteoid surface; OV, osteoid volume.

Supplemental Table 2. Bone histomorphometry at the metaphyses

	OV/BV, %	OS/BS, %	BV/TV, %	N.Oc/Bs, mm^{-1}
Vehicle	3.3 ± 0.9	19.0 ± 4.2	10.3 ± 4.6	1.0 ± 0.25
Lansoprazole	5.6 ± 2.1	30.0 ± 7.8	9.5 ± 2.9	0.70 ± 0.13
Statistical significance	$p < 0.04$	$p < 0.02$	not significant	$p < 0.05$

Means ± SD are indicated ($n = 8$ per group).

OV, osteoid volume; BV, bone volume; OS, osteoid surface; BS, bone surface; TV, tissue volume; N.Oc, osteoclast number.
